# Lipidomic diversity and proxy implications of archaea from cold seep sediments of the South China Sea

**DOI:** 10.3389/fmicb.2023.1241958

**Published:** 2023-10-19

**Authors:** Tingting Zhang, Wei He, Qianyong Liang, Fengfeng Zheng, Xi Xiao, Zhiyu Zeng, Jingzhuo Zhou, Wenyong Yao, Haodong Chen, Yuanqing Zhu, Jing Zhao, Yan Zheng, Chuanlun Zhang

**Affiliations:** ^1^Guangzhou Marine Geological Survey, China Geological Survey, Guangzhou, China; ^2^National Engineering Research Center of Gas Hydrate Exploration and Development, Guangzhou, China; ^3^East China Sea Ecological Center, Ministry of Natural Resources, Shanghai, China; ^4^Shenzhen Key Laboratory of Marine Archaea Geo-Omics, Department of Ocean Science and Engineering, Southern University of Science and Technology, Shenzhen, China; ^5^Southern Marine Science and Engineering Guangdong Laboratory (Guangzhou), Guangzhou, China; ^6^Key Laboratory of Coal Processing and Efficient Utilization of Ministry of Education, School of Chemical Engineering and Technology, China University of Mining and Technology, Xuzhou, China; ^7^State Key Laboratory of Organic Geochemistry, Guangzhou Institute of Geochemistry, Chinese Academy of Sciences, Guangzhou, China; ^8^Shanghai Sheshan National Geophysical Observatory, Shanghai, China; ^9^School of Environmental Science and Engineering, Southern University of Science and Technology, Shenzhen, China

**Keywords:** cold seeps, South China Sea, archaeal lipid biomarkers, ANMEs, methane index

## Abstract

Cold seeps on the continental margins are characterized by intense microbial activities that consume a large portion of methane by anaerobic methanotrophic archaea (ANME) through anaerobic oxidation of methane (AOM). Although ANMEs are known to contain unique ether lipids that may have an important function in marine carbon cycling, their full lipidomic profiles and functional distribution in particular cold-seep settings are still poorly characterized. Here, we combined the 16S rRNA gene sequencing and lipidomic approaches to analyze archaeal communities and their lipids in cold seep sediments with distinct methane supplies from the South China Sea. The archaeal community was dominated by ANME-1 in the moderate seepage area with strong methane emission. Low seepage area presented higher archaeal diversity covering Lokiarchaeia, Bathyarchaeia, and Thermoplasmata. A total of 55 core lipids (CLs) and intact polar lipids (IPLs) of archaea were identified, which included glycerol dialkyl glycerol tetraethers (GDGTs), hydroxy-GDGTs (OH-GDGTs), archaeol (AR), hydroxyarchaeol (OH-AR), and dihydroxyarchaeol (2OH-AR). Diverse polar headgroups constituted the archaeal IPLs. High concentrations of dissolved inorganic carbon (DIC) with depleted δ^13^C_DIC_ and high methane index (MI) values based on both CLs (MI_CL_) and IPLs (MI_IPL_) indicate that ANMEs were active in the moderate seepage area. The ANME-2 and ANME-3 clades were characterized by enhanced glycosidic and phosphoric diether lipids production, indicating their potential role in coupling carbon and phosphurus cycling in cold seep ecosystems. ANME-1, though representing a smaller proportion of total archaea than ANME-2 and ANME-3 in the low seepage area, showed a positive correlation with MI_IPL_, indicating a different mechanism contributing to the IPL-GDGT pool. This also suggests that MI_IPL_ could be a sensitive index to trace AOM activities performed by ANME-1. Overall, our study expands the understanding of the archaeal lipid composition in the cold seep and improves the application of MI using intact polar lipids that potentially link to extent ANME activities.

## Introduction

Methane is the second most important greenhouse gas that contributes to global warming at an increasing pace and has continually accumulated in the atmosphere over the past decades (Wuebbles, 2002; Saunois et al., 2020; Lauvaux et al., 2022). A large amount of methane in sediments of continental margins can emit to the surface and water column when the seabed is influenced by a variety of unstable geological factors (Boetius and Wenzhöfer, 2013). This continuously or intermittently occurring emission of methane on the seafloor forms methane plumes and various seabed structures like mud volcanoes, pockmarks, and carbonate chimneys (Paull et al., 1984; Ceramicola et al., 2018; Feng et al., 2018). These methane leaking areas form unique cold seep systems that often host prosperous ecosystems with vibrant macrofauna and microfauna (Suess, 2020).

Microbes like anaerobic methanotrophic archaea (ANME) in the cold seep sediments make use of methane by the sulfate-dependent anaerobic oxidation of methane (AOM; Hoehler et al., 1994; Hinrichs et al., 1999; Hinrichs and Boetius, 2002; Knittel et al., 2005). ANME and sulfate-reducing bacteria (SRB) dependent AOM provides methane-derived carbon (^13^C-depleted) and hydrogen sulfide to heterotrophic bacteria and endosymbiotic chemoautotrophic bacteria, which are the most important food source of large fauna such as vestimentiferan tube worms, vesicomyid clams, and bathymodiolid mussels typically found at most cold seeps in Pacific and Atlantic Oceans (Levin, 2005; Suess, 2020). Three major phylogenetically distinct groups of ANMEs have been discovered to participate in sulfate-dependent AOM, where ANME-1 and ANME-2 are usually associated with *Desulfosarcina-Desulfococcus* (DSS) group and ANME-3 with *Desulfobulbus* (DBB; Orphan et al., 2002; Zhang et al., 2002; Niemann et al., 2006; Losekann et al., 2007; Knittel and Boetius, 2009). AOM was also observed to be coupled with other electron acceptors, including nitrate (Raghoebarsing et al., 2006; Ettwig et al., 2010; Haroon et al., 2013; Arshad et al., 2015), nitrite (Ettwig et al., 2010), iron, and manganese (Beal et al., 2009; Egger et al., 2015; Ettwig et al., 2016).

Lipids are the basic components of cellular membranes, which are crucial for cells to maintain their integrity and individuality (Yang and Han, 2016; Villanueva et al., 2020). The core skeletons derived from membrane lipids (core lipids, CLs) of microbes are much more stable than DNA, which are ubiquitous in diverse environments (Zhang et al., 2002, 2004, 2006; Pearson et al., 2004, 2008; Zheng et al., 2019; Ma et al., 2020; Xiao et al., 2022). Glycerol dialkyl glycerol tetraethers (GDGTs) and archaeol (AR) are the most common core lipids of archaeal cell membrane (Supplementary Figure S1; Gambacorta et al., 1995). GDGTs are comprised by a series of compounds with 0–8 cyclopentane rings at the biphytane skeleton (GDGT-0 to GDGT-8). The unique GDGTs with four cyclopentane rings and one cyclohexane ring (crenarchaeol and crenarchaeol regioisomer) are considered as diagnostic biomarkers of ammonia-oxidizing archaea (AOA; Damsté et al., 2002; De La Torre et al., 2008). Those molecular fossils are useful biomarkers to trace ancient microbial activities and provide powerful qualitative and quantitative tools to reconstruct paleo-environment changes (Hinrichs et al., 2003; Schouten et al., 2013; Summons et al., 2021; Kim and Zhang, 2022, 2023; Rattanasriampaipong et al., 2022).

Our knowledge of the exact lipid profiles of ANMEs are still limited due to difficulties in isolating these archaeal groups. Alternatively, the combination of lipid composition and δ^13^C of lipids analysis, DNA-based sequencing of archaeal community, and multiple isotopic labeling approaches have been largely used to identified lipid biomarkers of these archaea. For example, the previous investigations of lipid profiles of ANMEs have revealed that ANME-1 generally synthesizes GDGTs and trace amount of diethers (AR), while ANME-2 and ANME-3 mainly produces AR and hydroxyarchaeol (OH-AR; Niemann and Elvert, 2008; Kurth et al., 2019). Therefore, the dominance of ANME-1 can be distinguished from ANME-2 and ANME-3 by the ration of OH-AR/AR (Niemann and Elvert, 2008). Further, the ANME-2 and ANME-3 can also be distinguished by tetramethylhexadecane (crocetane) and pentamethylicosane (PMI), where crocetane and its unsaturated homologs are found to be abundant in ANME-2, whereas PMI and its unsaturated homologs are ubiquitous in ANME-3 (Niemann and Elvert, 2008; Stadnitskaia et al., 2008). ^13^C-depleted crocetane and PMI are the most widespread and persistent lipid biomarkers of AOM related archaea, which have been widely detected in ancient seep deposits (Peckmann et al., 1999; Peckmann and Thiel, 2004; Peckmann et al., 2009). Moreover, the δ^13^C values of the lipids produced by ANME-2 exhibited a slightly larger offset from methane source compared to ANME-1 across diverse methane seep environments (Niemann and Elvert, 2008; Himmler et al., 2015; Miyajima et al., 2020), offering an alternative approach to discern the biological source of lipid biomarkers.

Intact polar lipids (IPLs) consisting of core lipid skeletons and various polar head groups are more labile lipid components that are considered as biomarkers for living microorganisms (Sturt et al., 2004). Hence, IPLs are widely used as a tool to estimate microbial biomass in soil, water column, and subsurface sedimentary environments (Lipp et al., 2008; Schubotz et al., 2009; Rethemeyer et al., 2010). The archaeal IPL profiles of ANMEs have been reported to have taxonomic specificity, however, the studies on their comprehensive lipidomic profiles still remain elusive. For example, Rossel et al. (2008, 2011) analyzed the composition of archaeal IPLs in various cold seep samples, and found that ANME-1 archaea mainly produced diglycosidic GDGTs while ANME-2 and ANME-3 synthesized intact polar (OH-) archaeol with glycosidic and phospho-headgroups or only phospho-headgroups, respectively (Rossel et al., 2008, 2011). Kurth et al. (2019) analyzed the lipid composition of *Ca. Methanoperedens* from the ANME-2d cluster, revealing the presence of mono (di) methyl-phosphatidyl ethanolamine and monopentose as headgroups that are rarely detected among ANME groups (Kurth et al., 2019).

The membrane compositions are diverse and distinct among different phylogenetic groups of archaea, which are likely to be regulated by the occurrence and activities of key genes and proteins involved in lipid biosynthesis (Zeng et al., 2019, 2022). The methane-impacted sediments can be distinguished from non-seep marine sediments by archaeal lipid composition. Typically, GDGT-0 and crenarchaeol were the most dominant archaeal membrane lipid compounds in non-seep marine sediments, which were mainly derived from Thaumarchaeota (now the class Nitrososphaeria) of overlay water columns. However, a high proportion of GDGT-1–3 were ubiquitously detected in methane-impacted sediment because of the contribution of the methanotrophic archaeal community thriving in the benthic sediments (Pancost et al., 2001; Blumenberg et al., 2004). Based on this observation and the δ^13^C values of those lipids, Zhang et al. (2011) proposed a molecular indicator “Methane Index (MI)” based on archaeal tetraethers to evaluate the contribution of methanotrophic archaea to the GDGT pool and assess the intensity of AOM (Zhang et al., 2011), which has been widely used to trace hydrate destabilization in Earth’s history (Zhang et al., 2011; Guan et al., 2016; Kim and Zhang, 2022).

Biogeochemical and geodynamic approaches have been performed on activities of cold seep at over 40 seep sites in northern and southern South China Sea (SCS; Feng et al., 2018). The Haima cold seep was first discovered in 2015 by ROV Haima and was one of the currently active seep sites in SCS (Liang et al., 2017). Gene amplification and sequencing analyses were widely employed to investigate microbial abundance, diversity, and function in SCS cold seep areas (Zhang et al., 2012, 2020; Wu et al., 2018; Cui et al., 2019; Zhuang et al., 2019; Li et al., 2020). Diverse archaeal groups have been identified in sediments from Haima cold seep area, including methane-metabolizing archaeal communities and Asgard superphylum (Niu et al., 2017; Zhang et al., 2020; Lu et al., 2021).

Lipid biomarkers and their δ^13^C values have been used to characterize microbial activities in cold seeps of southern SCS (Yu et al., 2008; Ge et al., 2011; Guan et al., 2018, 2022). Guan et al. (2018) analyzed methane-derived authigenic carbonates collected from Haima hydrocarbon seeps in SCS and detected diverse archaeal and bacterial biomarkers including crocetane, phytane, PMI, squalene, (OH-) archaeol, *n*-alkanes, fatty acids, hopanoids, and DAGEs, which possessed strongly depleted δ^13^C values (δ^13^C values as low as −126‰). The ^13^C-depleted biomarkers provided evidence for the dominance of ANME-1 with DSS in this area (Guan et al., 2018). Lipid biomarkers in depth profiles of sediments from Haima cold seep demonstrated that AOM was more active at the depths between 40 and 120 cm as revealed by extremely low δ^13^C_TIC_, δ^13^C_org_ values, and abundant ANME biomarkers (Guan et al., 2022).

Here, to better understand the active microbial communities involved in AOM and their full-scale lipid characteristics, we employed both 16S rRNA gene sequencing and lipidomic approaches to analyze cold seep sediments from SCS. We identified diverse lipids from cold seep in the northwestern SCS and performed a comprehensive study of archaeal IPLs in this area. Our results indicate that proxies based on core lipids and intact polar lipids can be used to trace the past and extant methane release and AOM activities, respectively.

## Materials and methods

### Sample collection and physicochemical analyses

**Figure 1:**
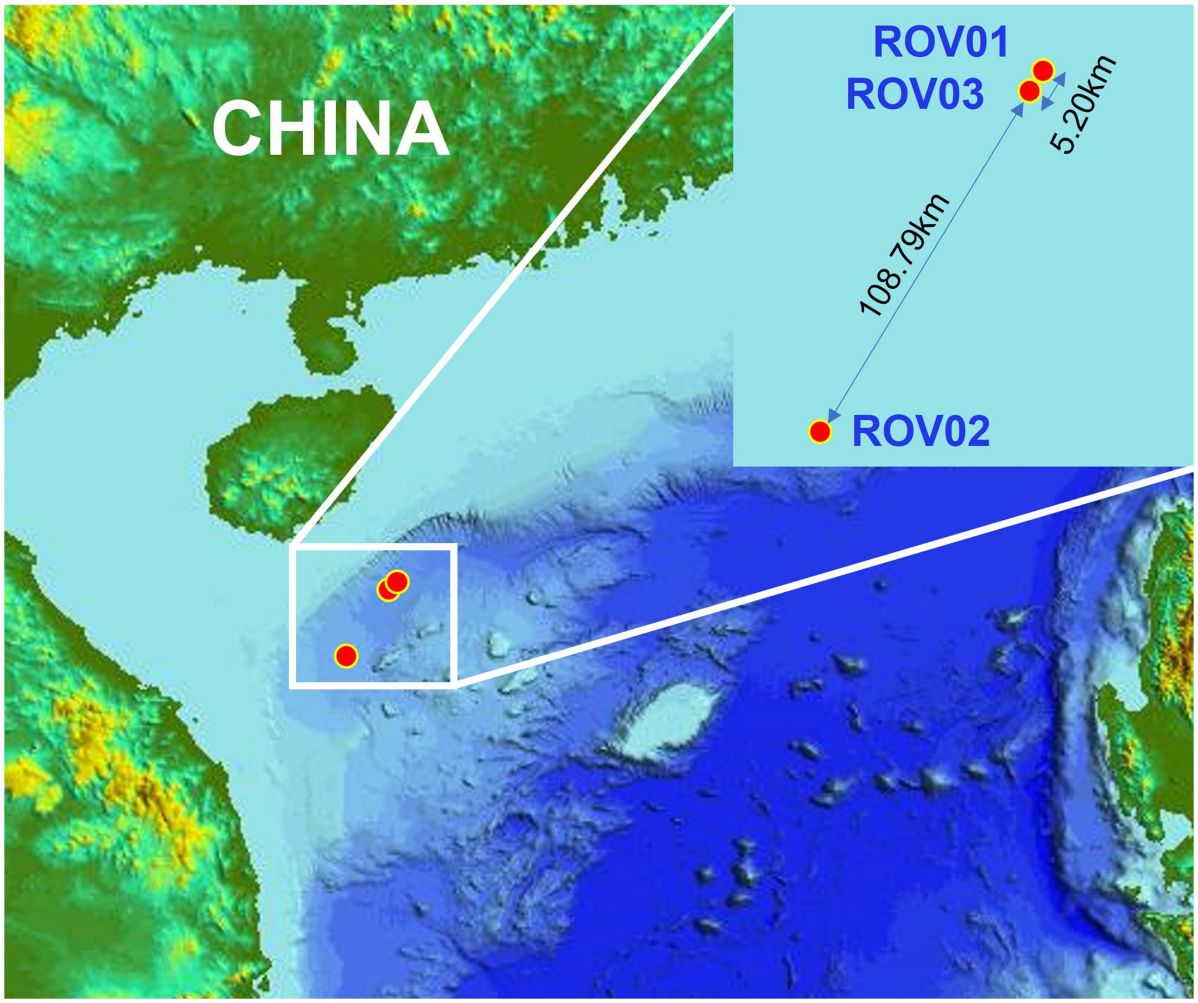
Map showing the study area and the cold seep sites in South China Sea.

Sediment samples were collected using the Haima ROV from different cold seeps on the northern Qiongdongnan Basin of the South China Sea during cruises HYDZ6-202002 and HYDZ6-202005. Push cores ROV01 and ROV03 were retrieved in May 2020 from the newly emerged cold seeps, which were discovered in 2018 (Zhang et al., 2020). The push core ROV01 was located in a dying cold seep, which was extensive and interspersed with an abundance of shells of dead *Calyptogena* clams (water depth: 1,736 m), exhibiting a strong smell of sulfide. The push core ROV03 was located 10 m beside a small mud mound (diameter: 10 m) colonized by dense populations of living *Calyptogena* clams (water depth: 1,715 m). Push core ROV02 was 2 m from the side of a pile of living *Calyptogena* clams (water depth: 1,395 m) in September 2020 from Haima cold seep (Figure 1).

Samples were collected every 10 cm depth interval onboard. The sediment subsamples for molecular analyses were then frozen at −20°C until further processing; the 10 mL sediment subsamples for methane analyses were transferred with a 5 mL cut-off syringe to a 20 mL Agilent bottle. The porewater samples for geochemical analyses were obtained using a Rhizon sampler (female luer, Rhizosphere, Netherlands). Total alkalinity (TA), Fe^2+^, Mn^2+^, PO_4_^3+^, NO_3_^−^, and NO_2_^−^ measurements were performed immediately by UV–visible spectrophotometer (Hitachi U-5100) onboard, following the “Specifications for oceanographic survey – Part 4: Survey of chemical parameters in sea water (GB/T 12763.4-2007).” For dissolved inorganic carbon (DIC), δ^13^C_DIC_ values and cation-anion concentrations (SO_4_^2−^, Cl^−^, Mg^2+^, Ca^2+^, K^+^, and Na^+^) determinations, porewater samples were kept at 4°C until measurement. For H_2_S measurements, 2 mL porewater samples were fixated with EDTA: NaOH (1,4) and kept at 4°C until analysis.

Methane concentrations were detected using a gas chromatograph (GC, Agilent 6850) equipped with flame ionization detector (FID). Carbon isotope values of methane were determined with a Gas Chromatography-Combustion-Isotope ratio Mass spectrometry (GC/C/IRMS). Methane gas was separated by gas chromatography and oxidized into carbon dioxide gas in the combustion furnace, and then introduced into an isotope mass spectrometer to determine the carbon isotope values of methane. Concentration and isotopic analyses of DIC, carbon and oxygen isotopes of carbonate were measured with an isotope ratio mass spectrometer (Thermo Delta V Advantage). Cation-anion concentrations (SO_4_^2−^, Cl^−^, Mg^2+^, Ca^2+^, K^+^, and Na^+^) were determined by an ion chromatography (ICS-1100, Thermo, United States) with conductance detector. H_2_S concentrations were detected by a Discrete Auto Analyzer (SmartChem200, Alliance, France).

### DNA extraction, sequencing, and bioinformatics

The total DNA of the sediments was extracted from about 0.3 g of sediment and purified using the E.Z.N.A.® soil DNA Kit. DNA concentrations were determined using a NanoDrop 2000 UV–vis spectrophotometer (Thermo Scientific, Wilmington, United States), and DNA qualities were verified by 1% agarose gel electrophoresis. The DNA amplification was performed by a thermocycler PCR system (GeneAmp 9700, ABI, United States) through the following process: 5 min of denaturation at 95°C; 35 cycles of sub-process constituting 30 s at 95°C, 30 s for annealing at appropriate temperature according to targeting bacterial and archaeal genes, and 1 min for elongation at 72°C successively; a final extension was at 72°C for 10 min. PCR reactions were performed in triplicate 20 μL mixture containing 10 μL of 2X Taq Plus Master Mix, 0.8 μL of each primer (5 μM), 7.4 μL of ddH_2_O, and 1 μL of template DNA. The archaeal 16S rDNA was amplified using barcoded primer sets Arch344F (5′-ACGGGGYGCAGCAGGCGCGA-3′; Raskin et al., 1994) and Arch915R (5′-GTGCTCCCCCGCCAATTCCT-3′; Stahl, 1991).

16S rDNA sequencing of target archaeal communities was performed with an Illumina MiSeq platform (Illumina, San Diego, United States) according to the standard protocols of Majorbio Bio-Pharm Technology Co. Ltd. (Shanghai, China). Low-quality sequences were demultiplexed, filtered by Trimmomatic, and merged by FLASH (Bolger et al., 2014) with the following criteria: (i) sequences having an average quality score < 20 over a 50 bp sliding window were truncated; (ii) sequences containing ambiguous bases were removed; and (iii) sequences having overlap of >10 bp were merged. The chimeric sequences were identified and removed using UCHIME (Edgar et al., 2011). High-quality sequences were clustered into operational taxonomic units (OTUs, 97% cut-off) using UPARSE (version 7.1, http://drive5.com/uparse/). Taxonomical assignments for representative sequences of OTUs were performed by RDP Classifier algorithm^1^ against the Silva (SSU132) 16S rDNA database using a confidence threshold of 70% (Quast et al., 2012).

### Lipid extraction and UPLC-MS analyses

Sediment samples were freeze-dried by a lyophilizer (BUCHI, Switzerland) and grinded by mortar and pestle. The powered sediments were used to extract the total lipid with the modified Bligh & Dyer lipid extraction procedure (Bligh and Dyer, 1959; White et al., 1979; Sturt et al., 2004). In detail, weighed samples were transferred to 50 mL Teflon centrifuge tubes and extracted twice with 19 mL solvent mixture of dichloromethane (DCM): methanol (MeOH): phosphate buffer at a ratio of 2:1:0.8 (v/v/v), and sequentially been treated with ultrasonic bath for 10 min. After sonication, samples were centrifuged and the supernatant was collected by glass flasks. Another two extractions were performed using solvent mixture of DCM: MeOH: trichloroacetic acid buffer (TCA) at a ratio of 2:1:0.8 (v/v/v) as described above. The supernatant of a sample was combined and 30 mL DCM and 30 mL ddH_2_O was added to make the final solvent ratio 1:1:0.9 (DCM: MeOH: H_2_O, v/v/v). After all, the mixture was separated, the organic phase (lower) was collected, and the remaining upper (aqueous) phase was re-extracted three times with DCM. The collected organic phase was condensed and dried under a gentle nitrogen flow. The total lipid was stored at −80°C until lipid quantification.

The total lipid extracts (TLEs) were dissolved in 1 mL methanol. An aliquot of the TLEs (0.5 mL) was dried under a gentle stream of nitrogen gas and re-dissolved in 200 μL methanol for lipid analysis. Lipids were analyzed on an ion mobility quadrupole time of flight mass spectrometer (IM-qTOF, Waters SYNAPT G2-Si), equipped with ACQUITY I-Class ultra-performance liquid chromatography (UPLC) system and an electrospray ionization source (ESI). The instrument parameters were optimized using β-L-gulosyl-phosphoglycerol dibiphytanyl glycerol tetraether (β-L-Gul-GDGT-PG, > 95%, Matreya LLC, United States). Compounds were separated using a C_18_-EXCEL column (2 μm, 2.1 mm × 150 mm, ACE) maintained at 45°C. 10 μL sample was injected and kept at 7°C during the whole run.

The chromatographic separation conditions were modified according to the method reported previously (Zhu et al., 2013). Briefly, the compounds were eluted with MeOH (Optima™ LC/MS grade, Fisher Scientific, PA, United States) as solvent A and 2-propanol (Optima™ LC/MS grade, Fisher Scientific, PA, United States) as solvent B, both amended with 0.04% formic acid (>99%, Optima™ LC/MS grade, Fisher Scientific, PA, United States) and 0.1% ammonia (25–30% NH_3_ basis, Sigma Aldrich, St. Louis, MO, United States). The elution gradient was set as 100% A for the first 5 min, then linearly increased phase B to 24% until 10 min, to 60% until 36 min, to 90% until 38 min, and maintained for 7 min. The mobile phase was shifted from 90% B to 100% A in 0.1 min and re-equilibrated until 55 min. The flow rate was maintained at 0.3 mL/min throughout the run.

The mass analyzer was performed in Resolution mode and the mass spectrometer detection of lipids was accomplished in FastDDA mode. The MS parameters were set as follows: Capillary voltage 2.5 kV, source temperature 120°C, sampling cone 45 V, cone gas flow 50 L/h, desolvation gas flow 800 L/h at 350°C, and nebulizer gas flow 6.5 bar. The ramped collision energy of the transfer cell was set as 10–55 V for low mass and 15–65 V for high mass, respectively. The mass range for MS^1^ was 100–2,000 Da, and the five most abundant ions were selected for MS^2^ experiment with a mass range of 50–2,000 Da. The mass accuracy was calibrated with sodium iodide initially and real-time monitored with leucine enkephalin ([M + H] ^+^ at m/z = 556.2771) as the calibration solution (scan time 0.2 s, interval 20 s).

Lipids were identified by their retention time, accurate mass, and diagnostic ions in MS^2^ spectra (Yoshinaga et al., 2011). Peak areas of identified compounds were integrated with MassLynx software (version 4.1) by combining three adduct forms ([M + H] ^+^, [M + NH_4_] ^+^, and [M + Na] ^+^), and the areas were then corrected for the amount of dry sediment weight (peak area/g; Rossel et al., 2008). The lack of commercial standards allowed only semi-quantitation based on peak areas without considering different response factors between different types of archaeal lipids.

### Proxy calculation and statistical analysis

Methane index (MI) was developed to estimate the activities of anaerobic methanotrophic archaea, which was calculated with the following equation (Zhang et al., 2011):


$$\mathrm{MI}=\frac{\left[\mathrm{GDGT}-1\right]+\left[\mathrm{GDGT}-2\right]+\left[\mathrm{GDGT}-3\right]}{\begin{array}{l} \left[\mathrm{GDGT}-1\right]+\left[\mathrm{GDGT}-2\right]+\left[\mathrm{GDGT}-3\right]\\ +\left[\mathrm{Crenarchaeol}\right]+\left[\mathrm{Crenarchaeol regioisomer}\right]. \end{array}}$$

Principal component analysis (PCA) was carried out by prcomp() function (default setting, except for scale. = TRUE) in the core package of R and visualized by ggbiplot (Vu, 2011). The box-and-wisker plot was generated by geom_boxplot() function in ggplot2 package (Wicham, 2016) and the geom_signif() function from the ggsignif package (Ahlmann-Eltze and Patil, 2021) was used to evaluate statistical differences between two samples areas (Wilcoxon Signed-Rank Test). All R functions and packages were implemented by R version 4.2.0.^2^

## Results

### Geochemical characteristics

**Figure 2:**
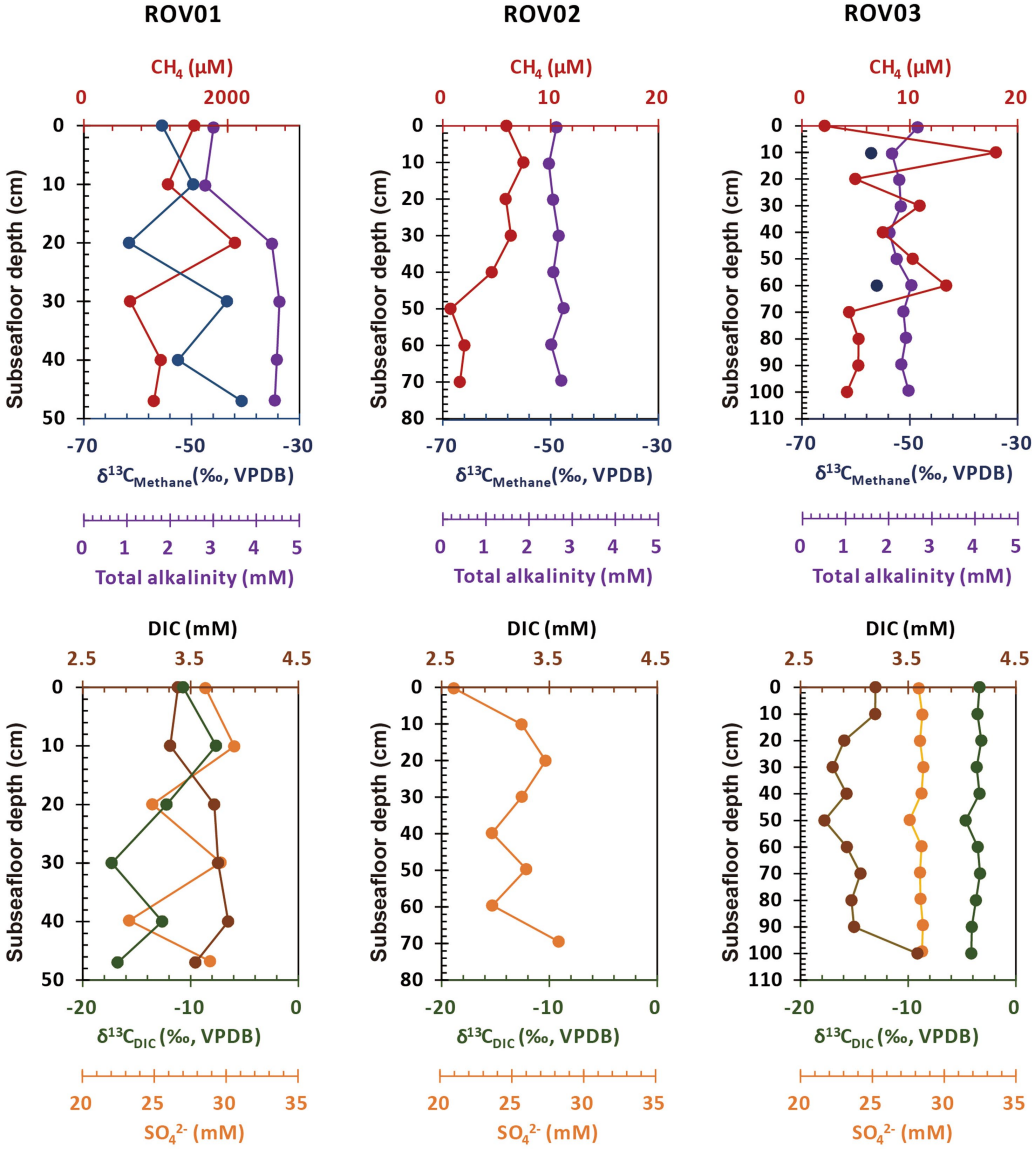
Concentration profiles of methane, δ^13^C_Methane_, sulfate, DIC, δ^13^C_DIC_, and total alkalinity in sediments from ROV01, ROV02, and ROV03. Distribution of CLs and IPLs.

Detailed geochemical features of three sediment cores were summarized in Supplementary Table S1. The methane concentration of core ROV01 varied from 647.38 μM at 30–40 centimeter below seafloor (cmbsf) to 2106.24 μM at 20–30 cmbsf, showed moderate impact of methane. However, this was likely to be underestimated because outgassing may be happened during sampling (Zhang et al., 2020). The ROV03 (from 2.1 μM at 0–10 cmbsf to 17.99 μM at 10–20 cmbsf) and ROV02 (from 0.79 μM at 50–60 cmbsf to 8.19 μM at 10–20 cmbsf) hold relatively lower methane concentration, which was 2–3 orders lower than core ROV01 (Figure 2; Supplementary Table S1). Here, we assigned sediment cores to moderate seepage (ROV01) and low seepage (ROV02 and ROV03) accordingly.

The δ^13^C_Methane_ was determined for the whole ROV01 profile and two relatively high methane concentration samples at ROV03 (10–20 cmbsf, −57.42 ‰; 60–70 cmbsf, −55.59 ‰). Overall, δ^13^C_Methane_ at ROV01 became less depleted as the depth increased. It was most depleted (−61.55 ‰) between 20 and 30 cmbsf and least depleted at the bottom layer (−40.67 ‰). Samples from core ROV01 also had high DIC concentrations, reaching a maximum (3.85 mM) at 40–47 cmbsf. ROV03 contained less DIC except for the bottom layer (100–112 cmbsf). Different from δ^13^C_Methane_, the lowest δ^13^C_DIC_ at ROV01 and ROV03 occurred at the middle and lower layers with δ^13^C_DIC_ of ROV01 changing more drastically than that in ROV03 (Figure 2; Supplementary Table S1).

The total alkalinity (TA) of the moderate seepage area (ROV01) also was higher than the low seepage areas (ROV02 and ROV03), especially at the deeper layer (> 20 cmbsf; Figure 2). Compared to ROV02 and ROV03, sediments from ROV01 exhibited strong smells of sulfide, and the highest sulfide concentration (1.07 mg/L) appeared at the 30–40 cmbsf region (Supplementary Table S1).

### Distribution of CLs and IPLs

**Figure 3:**
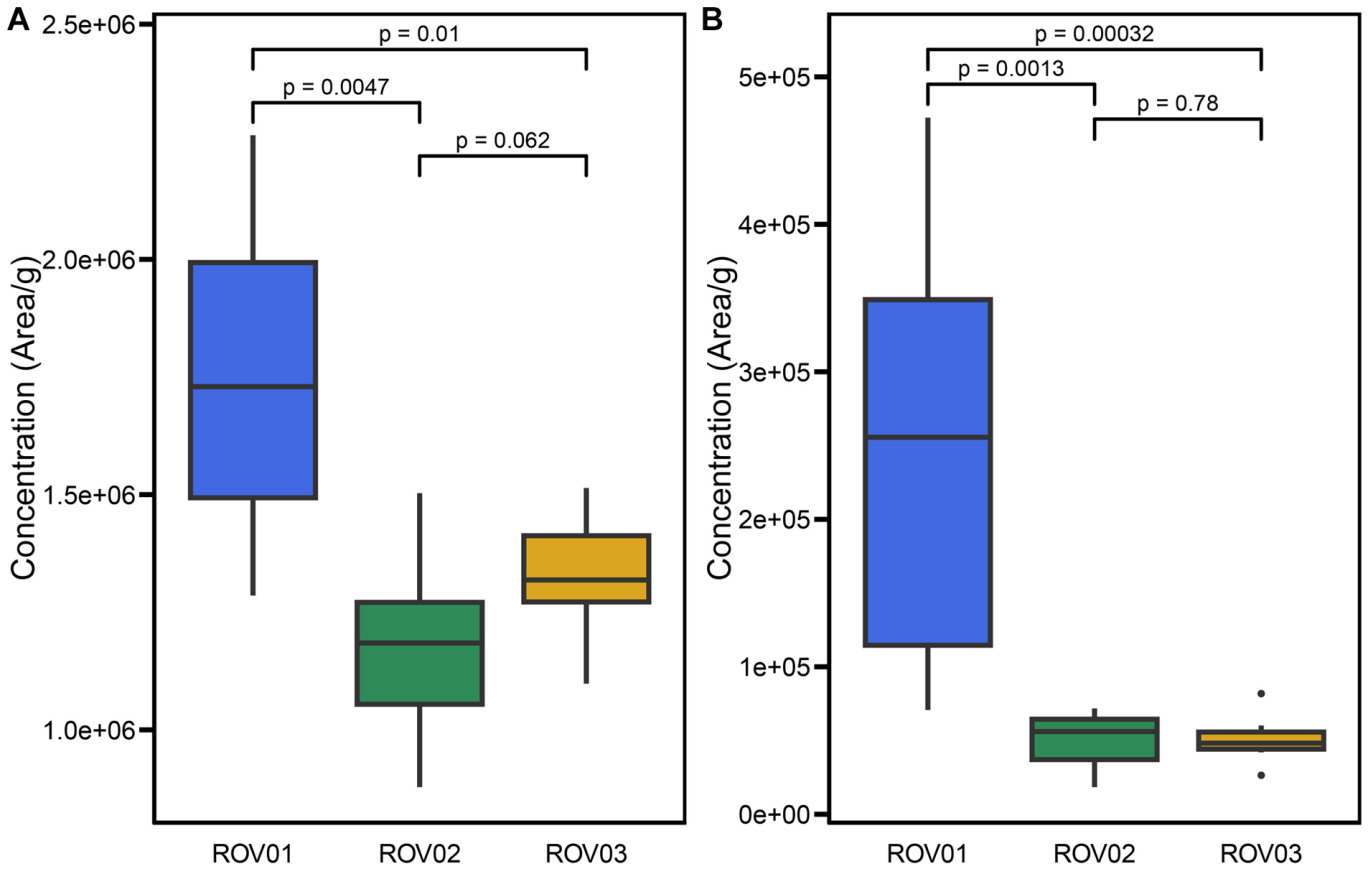
Box plots of **(A)** CLs and **(B)** IPLs concentration (areas/g, dry weight) in samples from ROV01 (blue), ROV02 (green), and ROV03 (gold). Significance level based on Wilcoxon Signed-Rank Test.

A total of 55 archaeal lipids were detected in the investigated samples, including 22 core lipids and 33 intact polar lipids (Supplementary Table S2, Supplementary Figures S2–S10). The relative abundance of core lipids from ROV01 ranged from 1.29 × 10^6^ area/g (ROV01-02) to 2.26 × 10^6^ area/g (ROV01-04), which was overall higher than that in at ROV02 (8.78 × 10^5^–1.50 × 10^6^ area/g) and ROV03 (1.10 × 10^6^–1.51 × 10^6^ area/g; Figure 3A; Table 1). Intact polar lipids at ROV01 were more abundant than those in ROV02 and ROV03 and accounted for a bigger proportion of the total lipids, especially in the sample ROV01-04 (17.26%; 30–40 cmbsf; Figure 3B; Table 1).

**Table 1:** Concentration (areas/g, dry weight) of archaeal lipids of sediments from ROV01, ROV02, and ROV03.

Sample ID	Total lipids (10^6^ areas/g)	CLs (10^6^ areas/g)	IPLs (10^5^ areas/g)
ROV01-01	1.66	1.59 (95.74%^#^)	0.71 (4.26%^#^)
ROV01-02	1.38	1.29 (92.91%)	0.98 (7.09%)
ROV01-03	2.38	2.03 (85.42%)	3.47 (14.58%)
ROV01-04	2.74	2.26 (82.74%)	4.72 (17.26%)
ROV01-05	2.22	1.87 (84.25%)	3.50 (15.75%)
ROV01-06	1.63	1.46 (89.91%)	1.64 (10.09%)
ROV02-01	0.90	0.88 (97.94%)	0.18 (2.06%)
ROV02-02	1.06	1.04 (97.95%)	0.22 (2.05%)
ROV02-03	1.10	1.06 (96.17%)	0.42 (3.83%)
ROV02-04	1.27	1.21 (95.30%)	0.60 (4.70%)
ROV02-05	1.49	1.42 (95.18%)	0.72 (4.82%)
ROV02-06	1.21	1.16 (95.66%)	0.53 (4.34%)
ROV02-07	1.57	1.50 (95.60%)	0.69 (4.40%)
ROV02-08	1.29	1.22 (95.10%)	0.63 (4.90%)
ROV03-01	1.37	1.32 (96.47%)	0.48 (3.53%)
ROV03-02	1.31	1.27 (96.80%)	0.42 (3.20%)
ROV03-03	1.30	1.27 (97.97%)	0.26 (2.03%)
ROV03-04	1.14	1.10 (96.16%)	0.44 (3.84%)
ROV03-05	1.32	1.27 (96.63%)	0.44 (3.37%)
ROV03-06	1.56	1.51 (97.03%)	0.46 (2.97%)
ROV03-07	1.47	1.42 (96.48%)	0.52 (3.52%)
ROV03-08	1.46	1.40 (96.16%)	0.56 (3.84%)
ROV03-09	1.42	1.36 (96.09%)	0.55 (3.91%)
ROV03-10	1.31	1.25 (95.41%)	0.60 (4.59%)
ROV03-11	1.55	1.47 (94.71%)	0.82 (5.29%)

^*^Concentrations of archaeal lipids are based on the sum peak area of molecules with [H]^+^, [NH_4_]^+^, and [Na]^+^ adduct forms in mass chromatograms. ^#^Numbers in brackets designate the relative abundance of CLs or IPLs for each sample.

**Figure 4:**
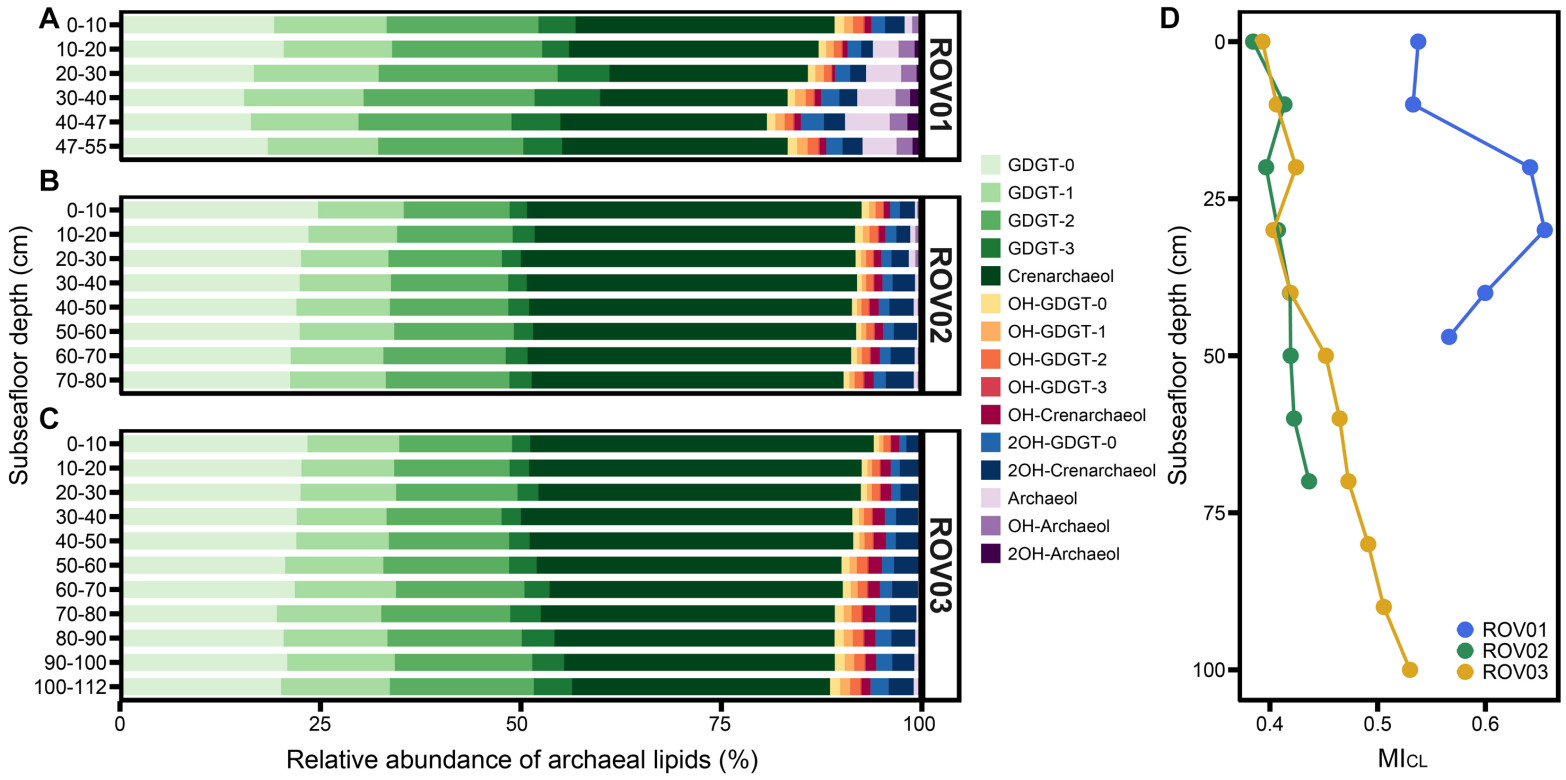
Relative abundance of CLs in samples from **(A)** ROV01, **(B)** ROV02, and **(C)** ROV03. Here, crenarchaeol and its isomers are represented by crenarchaeol, same with OH-crenarchaeol, 2OH-GDGT-0, and 2OH-crenarchaeol. **(D)** Methane index calculated with CLs (MI_CL_).

In all samples, the core lipids were dominated by GDGTs and contained low amounts of hydroxy-GDGTs (OH-GDGTs), AR, and OH-AR (Figures 4A–C). Core GDGTs and OH-GDGTs contained one to three cyclopentyl rings and four cyclopentyl rings plus one cyclohexyl ring. Core dihydroxy-GDGTs (2OH-GDGTs) with one to three cyclopentyl rings were not detected or below the detection limit (Supplementary Tables S2, S3). The proportion of diether in the moderate seepage area is higher than that in the low seepage area. In ROV01 and ROV03 the percentage of diethers increased with the depth (except for ROV01-06). In ROV02, the proportion of diethers was higher in the upper layers (10–30 cmbsf; Figure 4; Supplementary Figure S11A). The ratios of OH-AR to AR were lower than 1.1 in all samples, and the OH-AR/AR values in ROV02 were generally larger than those in ROV01 and ROV03 (Supplementary Table S3).

The methane index based on core GDGTs (MI_CL_) was calculated to reflect the past anaerobic methane oxidization carried out by ANME-1 groups (Supplementary Table S3). Samples from moderate seepage area owned higher MI_CL_ than that in low seepage area and reached their maximum at ROV01-04 (MI_CL_ = 0.66). MI_CL_ values increased with depth in both ROV02 and ROV03 and reached their maximum at the bottom layers (Figure 4D).

**Figure 5:**
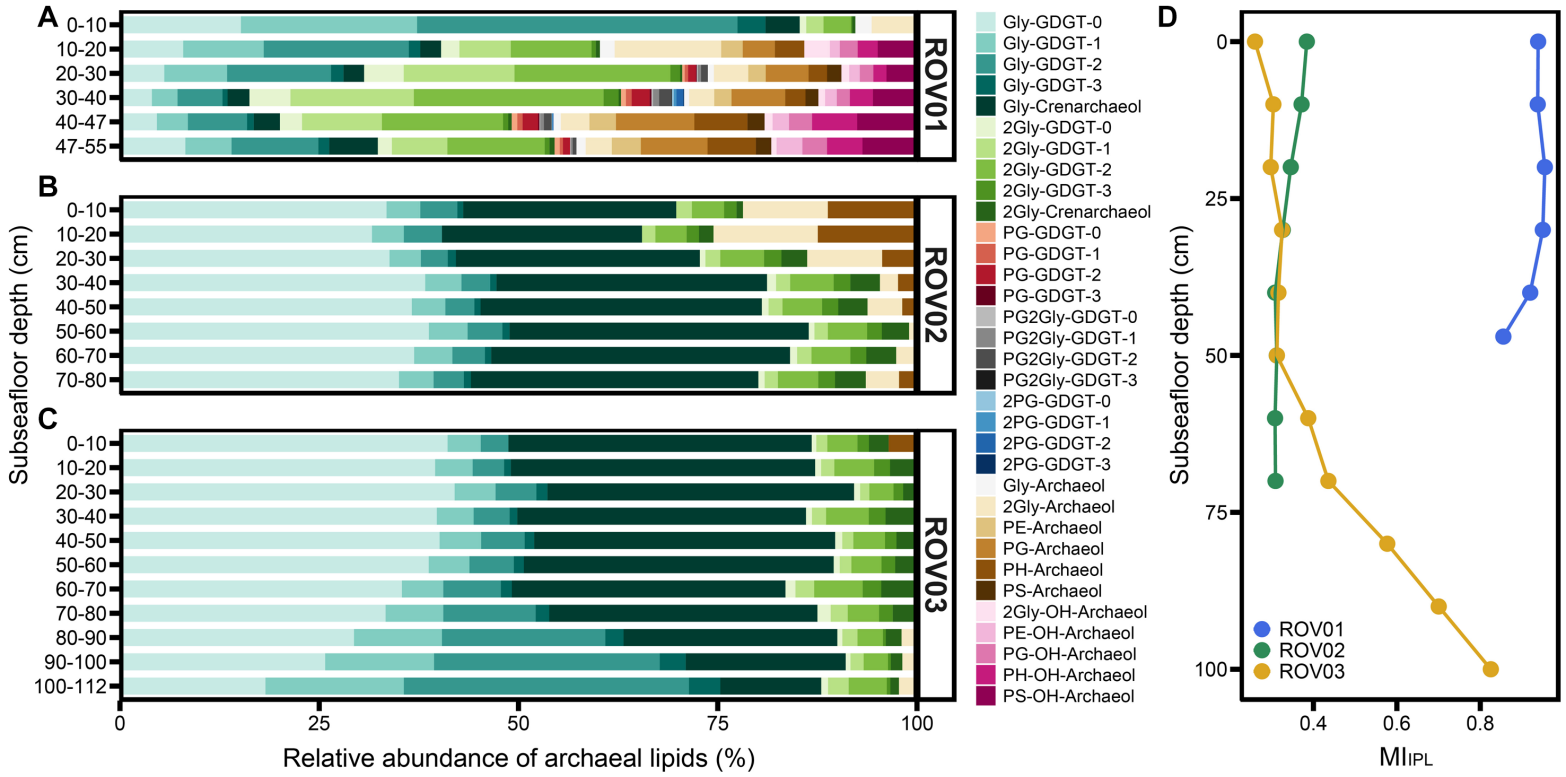
Relative abundance of IPLs in samples from **(A)** ROV01, **(B)** ROV02, and **(C)** ROV03. **(D)** Methane index calculated with IPLs (MI_IPL_). Archaeal community composition.

Intact polar lipids with various headgroups were detected in cold seep sediments (Supplementary Tables S2, S4). The detected headgroups included glycosyl (Gly), diglycosyl (2Gly), phosphatidylglycerol (PG), phosphatidylglyceroldiglycosyl (PG2Gly), diphosphatidylglycerol (2PG), phosphatidylethanolamine (PE), phosphatidylglycosyl (PH), and phosphatidylserine (PS; see Supplementary Figure S1 for structure information). Gly and 2Gly were detected with GDGT-0 to GDGT-3, crenarchaeol, archaeol, and OH-archaeol (except for Gly-OH-archaeol, which was missing). PG was connected with GDGT-0 to GDGT-3, archaeol, and OH-archaeol. PE, PH, and PS were only linked with archaeol and OH-archaeol. In addition, 2PG and PG2Gly only occurred with GDGT-0 to GDGT-3 (Figure 5; Supplementary Table S4).

Samples from moderate seepage area (ROV01) owned more diverse IPLs, where PG, 2PG, PG2Gly, PE, and PS were unique to ROV01. Besides, in ROV01, deeper layers possessed more diverse IPLs. The top layer (0–10 cmbsf) only contained glycosyl GDGTs (or archaeol) and diglycosyl GDGTs (or archaeol), and GDGTs with glycerol in the headgroup were also absent in the shallower layers (Figure 5A; Supplementary Table S4).

Similar to core lipids, intact polar lipids were dominated by GDGTs, while the diether/tetraether ratio of IPLs was different and much greater than that of CLs, especially at the deeper layer of ROV01 and the shallower layers of ROV02 (Supplementary Figure S11B). The IPL-OH-AR/IPL-AR ratios were consistently below 0.8 in all samples, resembling the patterns observed for CL-OH-AR/CL-AR (Supplementary Table S4). More specifically, the ratios of 2Gly-OH-AR to Gly-AR were the lowest among various polar head groups. Conversely, the PS-OH-AR/PS-AR values were significantly greater than other IPLs derived OH-AR/AR values (Supplementary Table S4).

The IPL methane index (MI_IPL_) showed extremely high values at ROV01 (MI_IPL_ > 0.85) and reached its maximum at 30–40 cmbsf (Figure 5D; Supplementary Table S4). MI_IPL_ values of ROV02 were lower than MI_CL_ values at this site and slightly dropped along the depth (Figures 4D, 5D). Similar to ML_CL_, MI_IPL_ values at ROV03 increased with depth and reached the maximum at the lowest layer, which was close to the MI_IPL_ values in ROV01 (Figure 5D; Supplementary Table S4).

### Archaeal community composition

**Figure 6:**
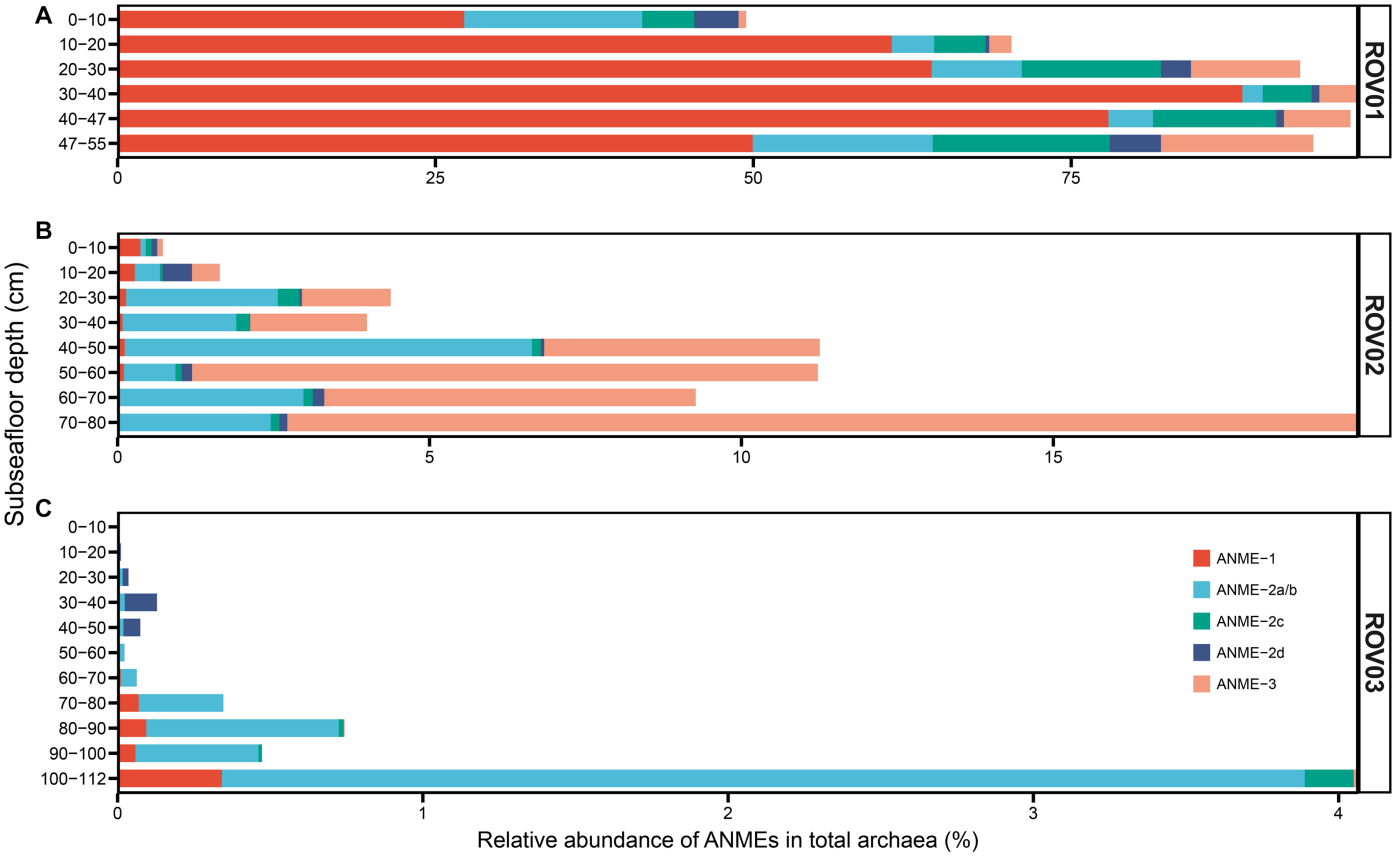
Relative abundance of ANMEs based on 16S rRNA gene sequencing data in **(A)** ROV01, **(B)** ROV02, and **(C)** ROV03.

ANME-1 and ANME-2 were the most abundant archaea in ROV01 and comprised over 50% of the total archaeal community (except for ROV01-01, 49.44%). Compared to ANME-2 and ANME-3, ANME-1 displayed a markedly prevailing presence. ANMEs increased proportionally with depth and reached the maximum at ROV01-04 (97.57%, Figure 6A; Supplementary Table S5), and the same was true for the ANME-1 group. ANEM-2 and ANME-3 were more abundant at the top layer and bottom layer of ROV01. Other major archaeal groups that thrived in ROV01 were Asgardarchaeota, Crenarchaeota, Nanoarchaeota, and Thermoplasmatota. It is worth noting that a number of archaea at the top layer in ROV01 cannot be classified (Supplementary Figure S12).

Principal component analysis (PCA) indicated that the archaeal community dominated in the moderate seepage area differed from that in the low seepage area (Supplementary Figure S13). Both ROV02 and ROV03 were dominated by Asgardarchaeota (Lokiarchaeia), Crenarchaeota (Bathyarchaeia), Nanoarchaeota, and Thermoplasmatota. Archaea at the top 10 cm layer in both cores differed from those at deeper layers (Supplementary Figures S12, S13). Nitrososphaeria (previously called Thaumarchaeota) was abundant at the top layer of ROV02 and ROV03, which was much rarer in deeper layer samples. Besides, Hydrothermarchaeia at ROV02-01 and Nanoarchaeia at ROV03-01 were also more abundant than those in deeper layer samples, respectively (Supplementary Figure S12).

Although both ROV02 and ROV03 came from low seepage areas, ROV02 possessed a relatively higher proportion of ANMEs (average 7.8%; highest at ROV02-08, 19.89%) than ROV03. ANMEs in samples from ROV02 mainly consist of ANME-2 and ANME-3, and a few ANME-1 occurred at the top layer while faded away as the depth increased (Figure 6B). By comparison, ANME-1 was more common at the bottom of ROV03 and reached its maximum at ROV03-11 (Figure 6C). Similar to ROV01, the proportion of ANMEs in both ROV02 and ROV03 increased with depth and reached the maximum at the bottom layer (19.89 and 4.06% respectively; Figure 6; Supplementary Table S5).

## Discussion

### Change of archaeal community composition responding to differential methane impact in sediment profiles of the South China Sea

Anaerobic archaeal methanotrophs (ANMEs) were the dominant archaeal groups in moderate seepage area (ROV01), constituting over 50% of the total archaeal community, which was in agreement with previous studies (Knittel and Boetius, 2009). The high and variable DIC concentrations and negative δ^13^C_DIC_ values along the depth at ROV01 indicated significant AOM activities by ANMEs (Figure 2). High-efficiency sulfate reduction coupled with AOM was simultaneously observed in the gradual decrease in sulfate concentration with depth and the detection of sulfide. However, ANMEs were detected with relatively low abundant in two low seepage areas (ROV02 and ROV03, average 7.8 and 0.54%, respectively), while other archaeal groups such as Lokiarchaeia and Bathyarchaeia were more abundant, implying that the supply of methane may have played a major role in shaping the ecological niche of archaea in cold seep areas (Niu et al., 2022). Additionally, higher methane concentrations were observed in the upper 40 cmbsf of ROV02 and 60 cmbsf of ROV03, suggesting that AOM might have occurred at greater depths, consistent with the increased abundance of ANMEs below these depths (Figure 6).

ANME-1 predominated over ANME-2 and ANME-3 in ROV01, with their abundance increasing with core depth, consistent with observations in other methane-rich sediments and microbial mats from Eel River Basin, Guaymas Basin, and Gulf of Mexico (Orcutt, 2002; Orphan et al., 2002; Teske et al., 2002; Rossel et al., 2011). Conversely, ANME-2 or ANME-3 were the major archaeal groups in other sites, such as Ha°kon Mosby Mud Volcano and Hydrate Ridge (Knittel et al., 2003, 2005; Losekann et al., 2007). Zhang et al. (2020) also reported that ANME-2 was more abundant than ANME-1 and ANME-3 at both the DNA and RNA levels in cold seeps in the northern South China Sea, which was close to our study sites (Zhang et al., 2020). In our study, ANME-2 and ANME-3 were significantly more abundant than ANME-1 in the low seepage areas (ROV02 and ROV03, Figure 6). Previous studies have indicated that ANME-2 is more widely distributed in areas with higher oxygen concentrations in bottom waters on a global scale, while ANME-1 is more prevalent in sediments with low oxygen levels and anoxic bottom waters (Knittel et al., 2005; Rossel et al., 2011). However, the oxygen level of bottom water in our study and Zhang et al. (2020) were not measured, further investigation is required to determine the factors governing their distribution in cold seep environments.

### The intact polar archaeal lipidome reflects variation of active ANME groups

The intact polar lipids (IPLs) are suggested to be more liable than their core components after the cell lysis and have been widely applied to trace the activity of living microbes (Sturt et al., 2004; Lipp et al., 2008). Our results indicated that the concentration of archaeal IPLs in the moderate seepage sediment (ROV01) was significantly higher than that in the other two low seepage sites, suggesting a substantial contribution from active benthic archaeal groups to the IPL pools. Furthermore, the proportion of IPLs was also relatively high in ROV01. The concentration of CLs was significantly higher in ROV01 than in other two low seepage cores, indicating a higher turnover rate of IPLs produced by active benthic archaeal groups and/or result of long-term accumulation.

In three cold seep sedimentary cores, we detected a diverse range of intact polar membrane lipids, primarily comprising diether (AR) and tetraether lipids (GDGTs) with various glycosyl and phosphatidyl head groups. The IPL-GDGTs were predominated by glycolipids, including Gly-GDGTs and 2Gly-GDGTs, while PG-GDGTs, 2PG-GDGTs, and PG-2Gly-GDGTs were found as minor lipids in some samples (ROV01), consistent with previous findings (Rossel et al., 2008, 2011). Prior investigations have revealed that ANME-1 dominated the microbial mat from a microbial reef in the Black Sea, with 2Gly-GDGTs accounting for over 99% of total archaeal IPLs (Rossel et al., 2008). Our results demonstrated that moderate seepage sediments (ROV01) contained much fewer 2Gly-GDGTs compared to sediments or mats from Black Sea. This discrepancy may, in part, be attributed to differences in the ionization efficiency of distinct mass spectrometer systems.

In contrast, Gly-GDGTs were the predominant archaeal lipids, with the highest abundance observed in the surface sample (0–10 cmbsf) of ROV01. Gly-GDGTs were also commonly found and dominate in Nitrososphaeria cultures, but the abundance of Nitrososphaeria in the surface sediment was very low, indicating a potential contribution of Gly-GDGTs from planktonic Nitrososphaeria. However, Gly-GDGTs with 1–3 cyclopentane rings dominated the archaeal IPL pool instead of thaumarchaeal characteristic Gly-crenarchaeol (Elling et al., 2017). In fact, the predominance of GDGTs-1, -2, and -3 is a characteristic lipid pattern of ANME-1 (Rossel et al., 2008; Zhang et al., 2011). Based on the observation that ANME-1 overwhelmingly dominated in ROV01, our results suggested that Gly-GDGTs were likely primarily produced by ANME-1 rather than by Nitrososphaeria.

Furthermore, although ANME-2 and ANME-3 groups have been reported to mainly synthesize phosphate-based polar AR and OH-AR (Rossel et al., 2008; Knittel and Boetius, 2009), ANME-2c/d groups have also been found capable of producing a few GDGTs and OH-GDGTs (Elvert et al., 2005; Kurth et al., 2019). Hence, ANME-2c/d groups could also serve as additional microbial sources of Gly-GDGTs (Elvert et al., 2005; Kurth et al., 2019), which contributed to 9.65% on average of archaea in ROV01. Other archaea (e.g., Lokiarchaeia, Bathyarchaeia, and Nanoarchaeia) exhibited very low abundances (Supplementary Figure S12; Supplementary Table S5) and may exert a minor impact to the Gly-GDGTs.

Unlike ROV01, Gly-GDGTs in ROV02 and ROV03 mainly consisted of Gly-GDGT-0 and Gly-crenarchaeol, while 2Gly-GDGTs were dominated by 2Gly-GDGT-2 and 2Gly-crenarchaeol (Figure 5). This characterization was similar to that of IPL-GDGTs in the water column sample from the South China Sea, suggesting that the IPL-GDGTs in ROV02 and ROV03 sediments likely originated from the water column (Dong et al., 2018; Wu et al., 2019). Actually, some investigations have indicated that GDGTs with glycosidic headgroups preserved notably longer than phosphorous headgroups GDGTs, and some sedimentary glycosidic GDGTs could originated from upper water column (Schouten et al., 2010; Logemann et al., 2011; Lengger et al., 2014; Dong et al., 2018; Wu et al., 2019). An exception occurred in the deeper layers of ROV03, where the abundance of Gly-GDGT-1 and Gly-GDGT-2 significantly increased, even exceeding the contents of Gly-GDGT-0 and Gly-crenarchaeol (Figure 5). This suggested that the IPL-GDGTs produced by some local archaeal groups, such as ANME-1, gradually increased and significantly influenced the composition of the overall IPL-GDGT pool in the deep layers of ROV03.

The archaeol lipids with phosphatidyl headgroups, including PS-, PE-, PI-, and PG-(OH)archaeol, were abundant in the moderate seepage area. These lipids were previously found to be abundant in environments dominated by ANME-2 and ANME-3, which was consistent with our observations in ROV01 and reflected intensive ANME-2 and ANME-3 activities (Rossel et al., 2008, 2011; Yoshinaga et al., 2011; Kurth et al., 2019). ANME-2d were reported to contain pentose, monomethyl phosphatidyl ethanolamine (MMPE) and dimethyl phosphatidyl ethanolamine (DMPE) head groups (Kurth et al., 2019), which, however, were not detected in this study. This may due to the low abundance of ANME-2d group in our samples (Figure 6). Although the top layer of ROV01 had similar ANME-2 and ANME-3 composition to other layers, intact polar OH-AR and phosphate headgroups were missing (Figure 5A), implying that the composition of archaeal species is not the sole determinant of lipid patterns, and environmental factors may override genetic control on the lipid distribution.

Although ANME-1 has also been found to produce a few diethers (Blumenberg et al., 2004), prior research on lipids associated with cold seeps has shown that environments dominated by ANME-2 or ANME-3 typically exhibit higher OH-AR/AR ratios compared to those dominated by ANME-1 (Blumenberg et al., 2004; Elvert et al., 2005). In general, OH-AR/AR values range from 0 to 0.8 in ANME-1 dominated environments typically, but from 1.1 to 5.5 in ANME-2 or ANME-3 dominated environments (Niemann and Elvert, 2008). However, exceptions have been also observed, for instance, in a sample collected from the Northwestern Black Sea, Dniepr area, as reported by Rossel et al. (2008), the ratio of ANME-1 to ANME-2 was 35/65, indicating a prevalence of ANME-2 in this sample. Intriguingly, the archaeal lipid with the highest abundance in this sample was PG-AR (35%), while the sum of PS-OH-AR, 2Gly-OH-AR, and PE-OH-AR accounted for only 4% (Rossel et al., 2008). In the case of ROV01, ANME-1 were overwhelmingly abundant than ANME-2 and ANME-3, and both CLs derived OH-AR/AR and IPLs derived OH-AR/AR values were lower than 1.1 (Supplementary Tables S3, S4).

We also examined the OH-AR/AR ratios of diether lipids with different polar head groups (Supplementary Table S4). The ratio of 2Gly-OH-AR/2Gly-AR was the lowest among all polar head groups. Whereas PS derived OH-AR/AR values displayed an obvious ANME-2 characteristic (Supplementary Table S4). The varying degrees of hydroxylation of PE-, PH-, and PS-archaeol which were primarily produced by ANME-2 and ANME-3 could result from either the substrate preference of lipid synthases or environmental stresses, however, this need to be further investigated.

A small amount of IPL-AR was also detected in ROV02 and ROV03, primarily composed of 2Gly-AR and PH-AR. The relative abundance of these IPL-ARs decreased with depth in ROV02. In contrast, PH-AR was solely observed in the surface layer, while 2Gly-AR was exclusively present in the lowermost layers in ROV03 (Figure 5). These trends differed significantly from the changes observed in the abundance of ANME-2 and ANME-3 in these areas (Figure 6). This suggests that the IPL-ARs in ROV02 and ROV03 may primarily originate from non-ANME groups, such as Nitrososphaeria.

Previous reports have indeed suggested that Nitrososphaeria possesses the capability to produce glycosidic and phosphohexose ARs, albeit these IPL-ARs represent only a minor fraction of the total IPLs in Nitrososphaeria (Elling et al., 2014, 2017). Furthermore, the distribution profile of Nitrososphaeria, with higher abundance in the surface layer and lower abundance in the deep layer, aligns with the distribution profile of 2Gly-AR and PH-AR in the samples (Figure 6; Supplementary Figure S12). This further strengthens the hypothesis that Nitrososphaeria could be a potential biological source of IPL-ARs in ROV02 and ROV03. Simultaneously, we cannot dismiss the possibility that these IPL-ARs originated from Nitrososphaeria in the upper water column due to the higher IPL-ARs abundance in the shallow layer.

### Methane index tracks AOM activities in the cold seep

The proposed Methane index (MI; Zhang et al., 2011) has been widely utilized to track hydrate-impacted environments (Zhang et al., 2011; Guan et al., 2016; Kim and Zhang, 2022). The methane impacted sediments typically exhibited relatively high MI values, often exceeding 0.3–0.5, along with depleted δ^13^C of GDGTs (Zhang et al., 2011). Kim and Zhang (2023) further explored the quantitative relationship between MI and methane diffusive flux, demonstrating a strong association between high MI values and elevated methane diffusive fluxes in sediments. Our results also indicated that MI a promising tool for tracking AOM activities. The high values of both MI_CL_ and MI_IPL_ (> 0.3–0.5) in our study areas indicated that AOM might have sustained for a long time and it is likely ongoing in these three areas. Compared to low seepage areas, moderate seepage sediments exhibited higher MI values (Figures 4D, 5D), implying a more intense methane impact at this site. The highest MI_CL_ and MI_IPL_ values were observed within the interval between 20 and 40 cmbsf in ROV01, supporting an anomalously strong AOM activities at this depth (Figures 4D, 5D).

The decrease in MI_IPL_ values with depth matched the decreasing proportion of the ANME-1 group in ROV02. However, MI_CL_ has an opposite trend to MI_IPL_ (Figure 4D; Supplementary Table S3) in this area, indicating that there might have been a higher proportion of the ANME-1 group at the deeper layers in ROV02. The both increases in MI_CL_ and MI_IPL_ in ROV03 were consistent with the fact that the proportion of the ANME-1 group increased at the deeper layers (Figures 4D, 5D). The extremely low proportion of the ANME-1 group (≤0.37% in ROV02, ≤0.34% in ROV03) in contrast to high MI_IPL_ values in ROV02 and ROV03 indicates that MI_IPL_ may be highly sensitive and can be applied in environments where ANME-1 group does not dominate the microbial community, and the methane flux is extremely low.

## Conclusion

The emission of methane gas shapes unique microbial communities in cold seep habitats. In this study, we characterized the archaea community and its lipid features of three sediment cores from the cold seep area in the South China Sea with 16S rRNA gene sequencing and lipidomics based approaches. Our data show that ANMEs were the most abundant archaea group in the moderate seepage area while Lokiarchaeia and Bathyarchaeia dominated the low seepage area. The MI values based on CL-GDGTs and IPL-GDGTs in the moderate seepage area were both higher than those in low seepage area which indicated strong AOM in methane-rich area. MI_IPL_ is a highly sensitive index that can be used to infer AOM activities carried out by the extremely low abundance of the ANME-1 group. The comparison between MI_CL_ and MI_IPL_ provides a potential way to study the evolution of microbial communities in ANME-1 thrived environments. Although lipid biomarkers are effective tools to reflect microbial activities and help us to reconstruct the paleoenvironment, more comprehensive and accurate identification of lipids in environment samples is still challenging. A more complete lipid investigation with high throughput archaeal lipid identification needs to be carried out.

## Data availability statement

The datasets presented in this study can be found in online repositories. The names of the repository/repositories and accession number(s) can be found at: NCBI—PRJNA985914.

## Author contributions

WH, TZ, and FZ designed the study. TZ did sampling on board and performed bioinformatics analysis. XX and HC finished the geochemical experiments. ZZ, JZho, and WY performed the lipid extraction and assisted lipidomics data analysis. WH and TZ analyzed the data, prepared the figures, and wrote the drift manuscript. QL, FZ, YuZ, JZha, YaZ and CZ edited and approved the final manuscript. All authors contributed to the article and approved the submitted version.

## Funding

Financial supports for this research were provided by the National Natural Science Foundation of China (No. 42003063 to FZ and No. 42276087 to QL), the Marine Geological Survey Program of China Geological Survey (No. DD20221706 and DD20230065 to QL), the Guangdong Basic and Applied Basic Research Foundation (No. 2019B030302004 to QL), the Stable Support Plan Program of Shenzhen Natural Science Fund (No. 20200925173954005 to CZ), the Shenzhen Key Laboratory of Marine Archaea Geo-Omics, Southern University of Science and Technology (No. ZDSYS201802081843490 to CZ), and the Special Funds for the Cultivation of Guangdong College Students’ Scientific and Technological Innovation (“Climbing Program” Special Funds, No. pdjh2021b0441 to WH).

## Footnotes

^1^http://rdp.cme.msu.edu/

^2^http://www.r-project.org
