# Supplementary material for: Lipidomic diversity and proxy implications of archaea from cold seep sediments of the South China Sea
Source: Front Microbiol. 2023 Oct 19;14:1241958. doi: 10.3389/fmicb.2023.1241958 (PMC10635418; doi:10.3389/fmicb.2023.1241958)
Supplement: Supplementary file 2 [file Data_Sheet_1.docx]

Lipidomic diversity and proxy implications of archaea from cold seep sediments of the South China Sea

Tingting Zhang, Wei He, Qianyong Liang, Fengfeng Zheng, Xi Xiao, Zhiyu Zeng, Jingzhuo Zhou, Wenyong Yao, Haodong Chen, Yuanqing Zhu, Jing Zhao, Yan Zheng, Chuanlun Zhang

email: tomlqy@163.com (Q. Liang); zhengff@sustech.edu.cn (F. Zheng)

Supplementary Material

# Supplementary Figure

**
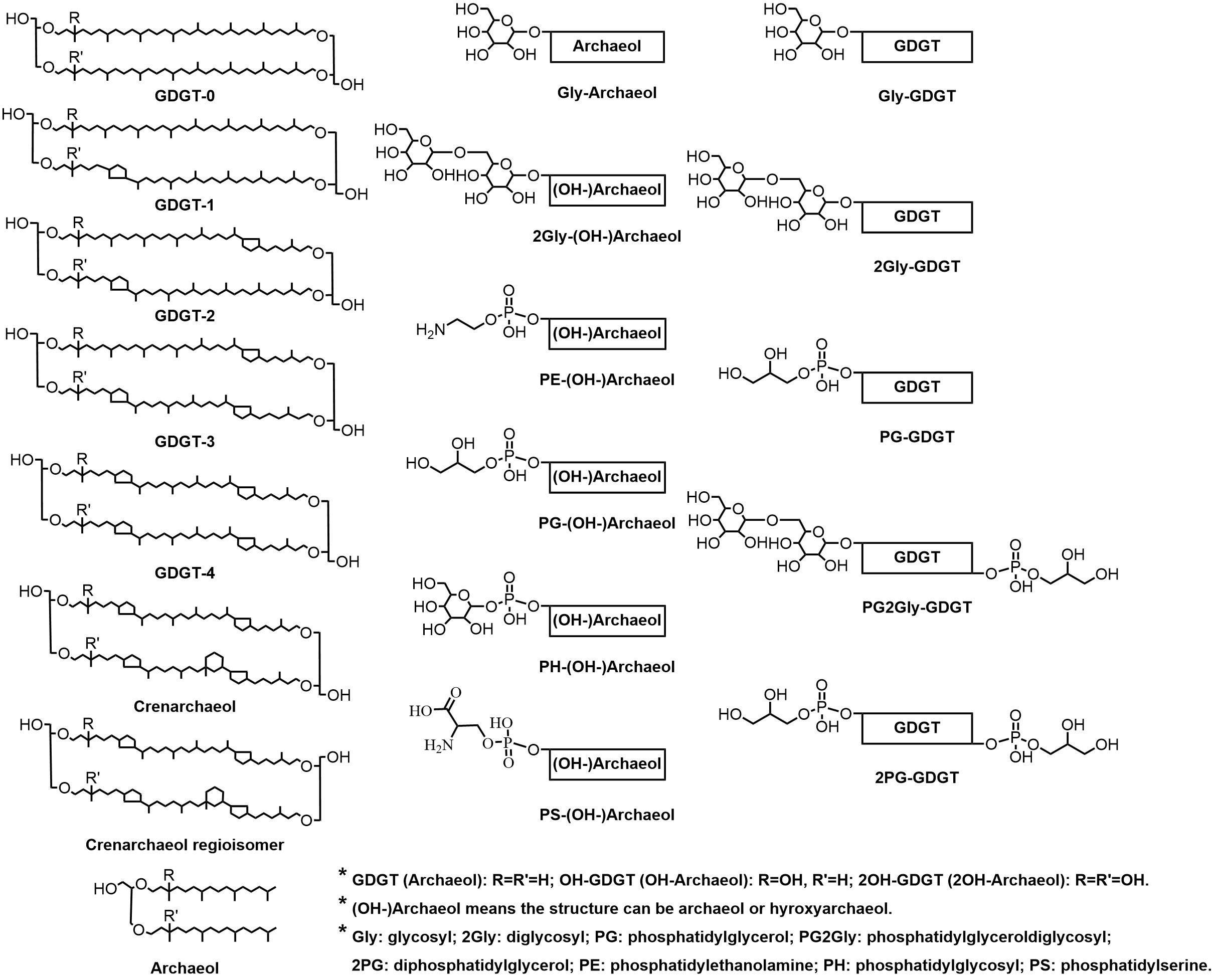
**

**Figure S1.** Structures of archaeal CLs and IPLs identified in cold seep sediments of this study.


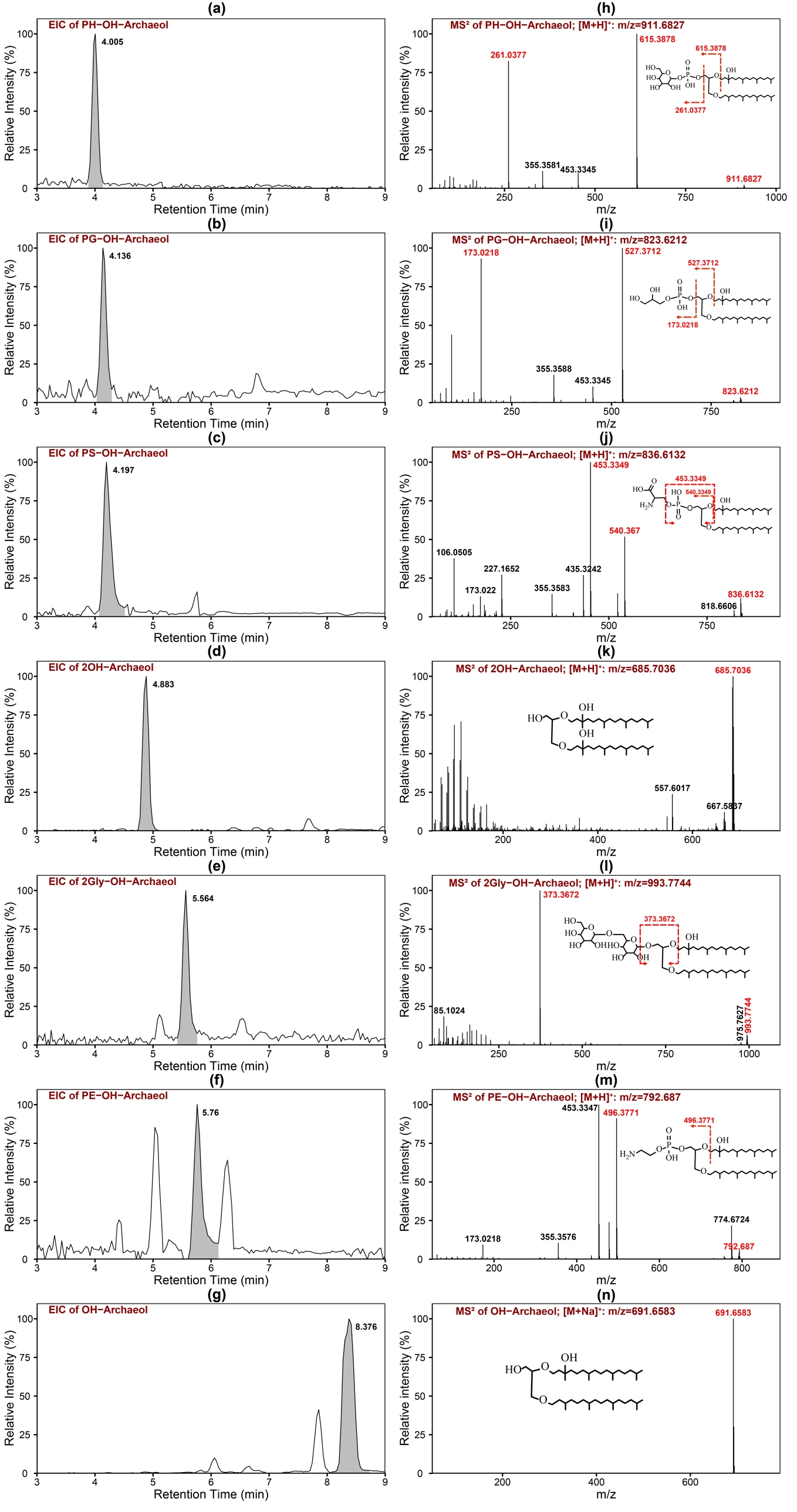


Figure S2. (a)-(g) Extract ion chromatograms (EICs, [M+H]^+^+[M+NH_4_]^+^+[M+Na]^+^) of core and intact polar hydroxyarchaeol; (h)-(n) MS^2^ spectra of core and intact polar hydroxyarchaeol, representative precursor ion and diagnostic product ions are highlight in red.


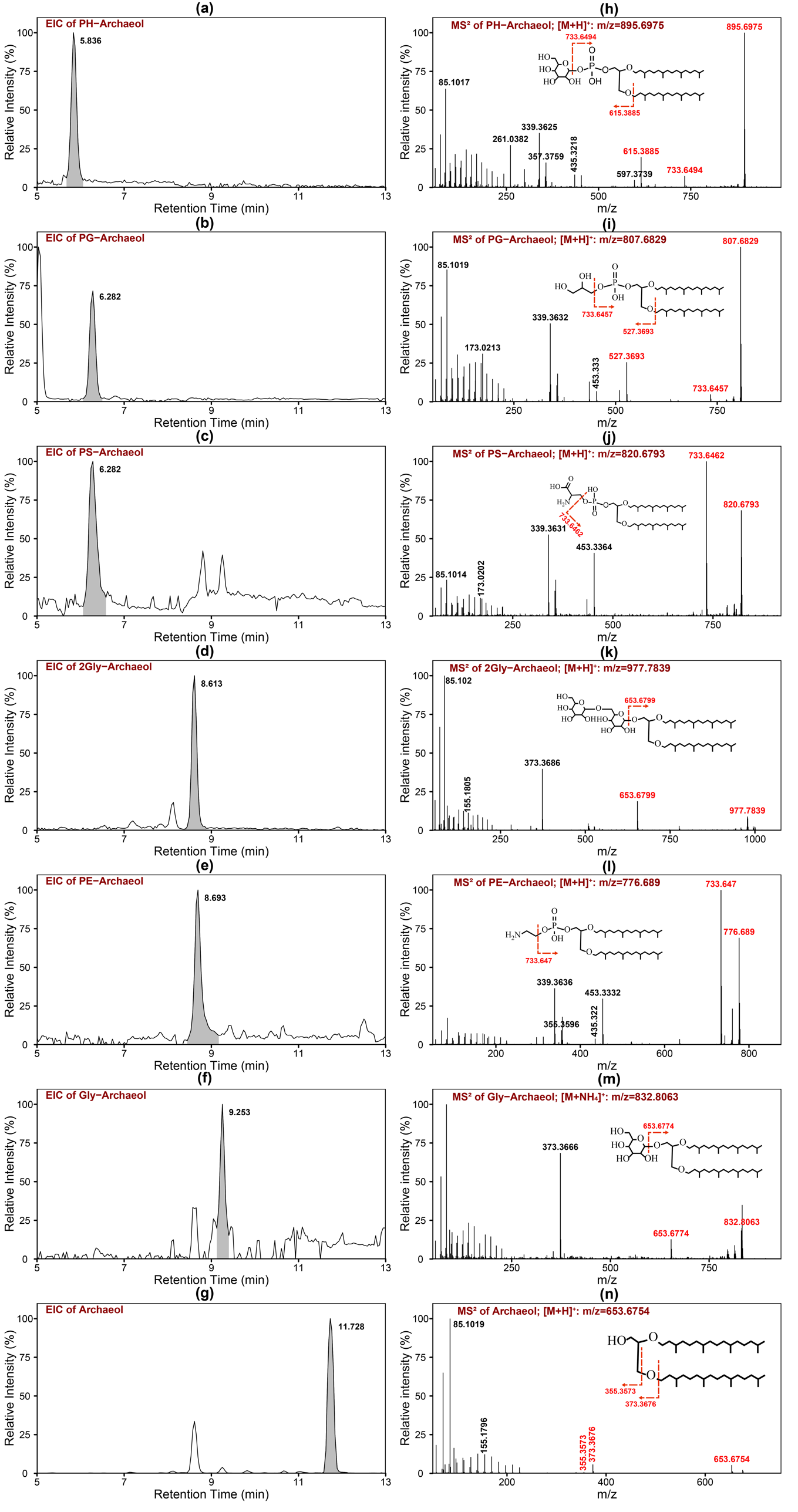


Figure S3. (a)-(g) Extract ion chromatograms (EICs, [M+H]^+^+[M+NH4]^+^+[M+Na]^+^) of core and intact polar archaeol; (h)-(n) MS^2^ spectra of core and intact polar archaeol, representative precursor ion and diagnostic product ions are highlight in red.


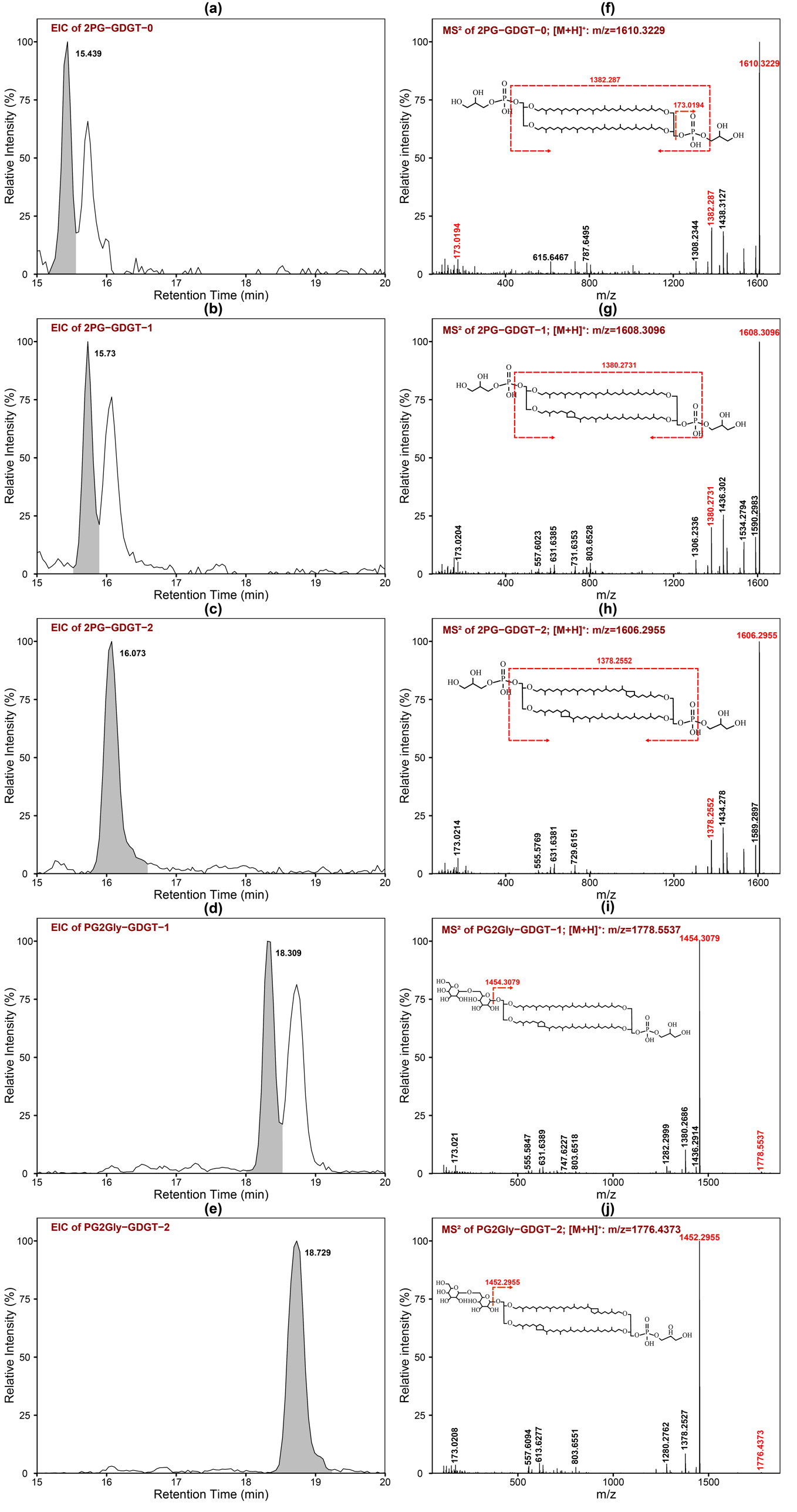


Figure S4. (a)-(e) Extract ion chromatograms (EICs, [M+H]^+^+[M+NH4]^+^+[M+Na]^+^) of 2PG-GDGTs and PG2Gly-GDGTs; (f)-(j) MS^2^ spectra of 2PG-GDGTs and PG2Gly-GDGTs, representative precursor ion and diagnostic product ions are highlight in red.


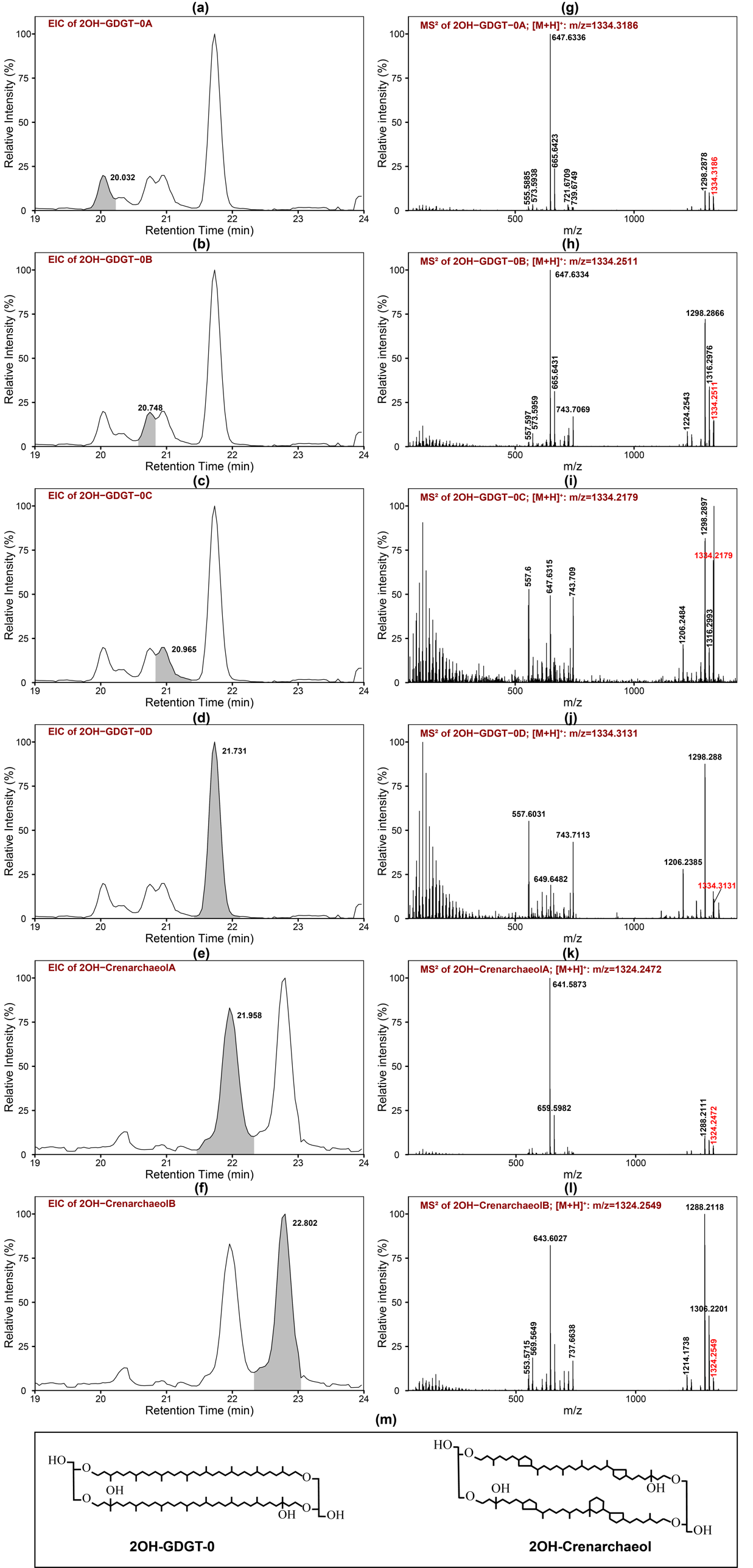


Figure S5. (a)-(f) Extract ion chromatograms (EICs, [M+H]^+^+[M+NH4]^+^+[M+Na]^+^) of 2OH-GDGTs; (g)-(l) MS^2^ spectra of 2OH-GDGTs, representative precursor ion is highlight in red; (m) Structures of 2OH-GDGTs. Because the elution time of 2OH-GDGTs and their isomers is unclear, the structures related to the spectra are not shown here.


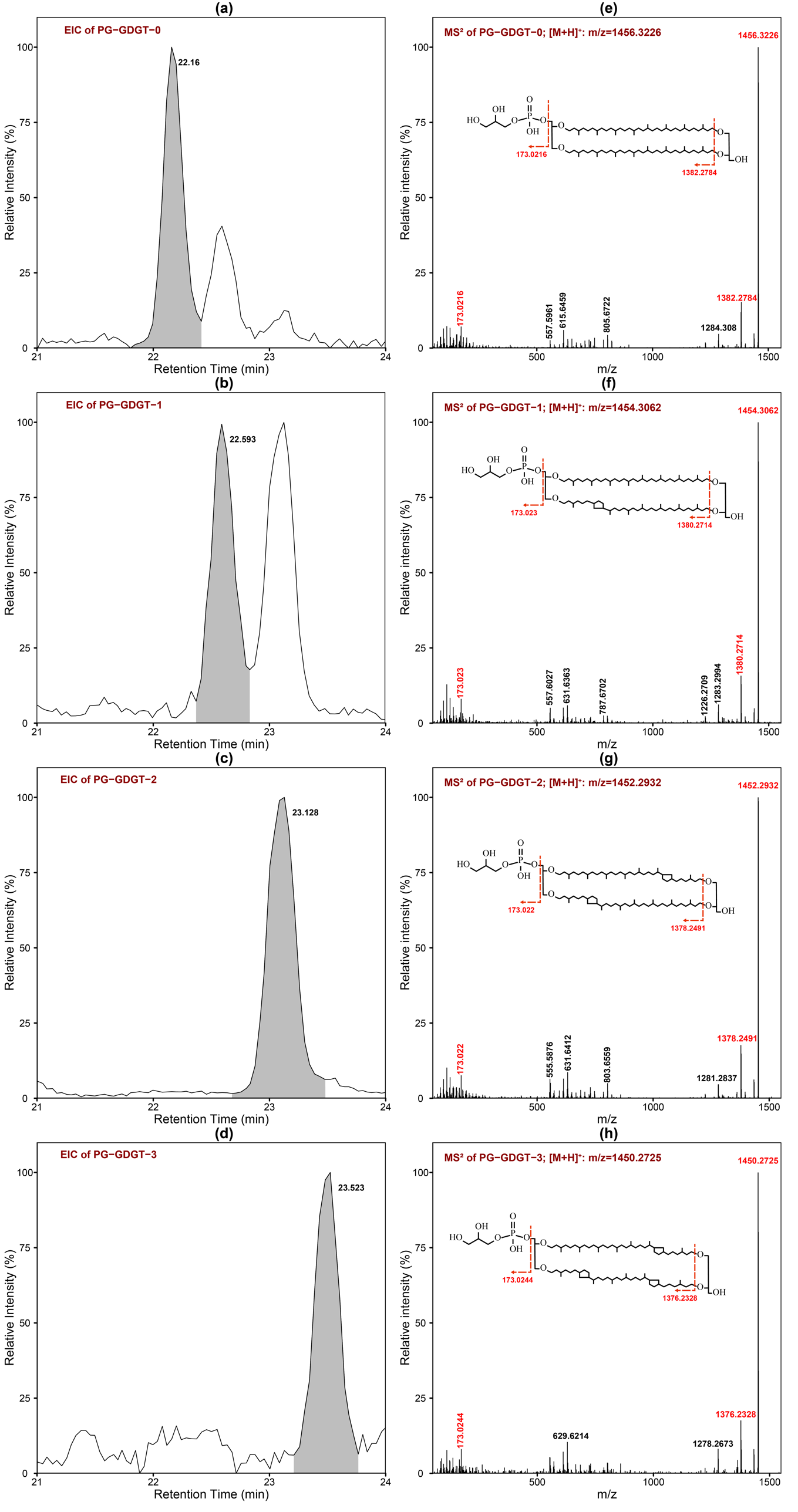


Figure S6. (a)-(d) Extract ion chromatograms (EICs, [M+H]^+^+[M+NH4]^+^+[M+Na]^+^) of PG-GDGTs; (e)-(h) MS^2^ spectra of PG-GDGTs, representative precursor ion and diagnostic product ions are highlight in red.


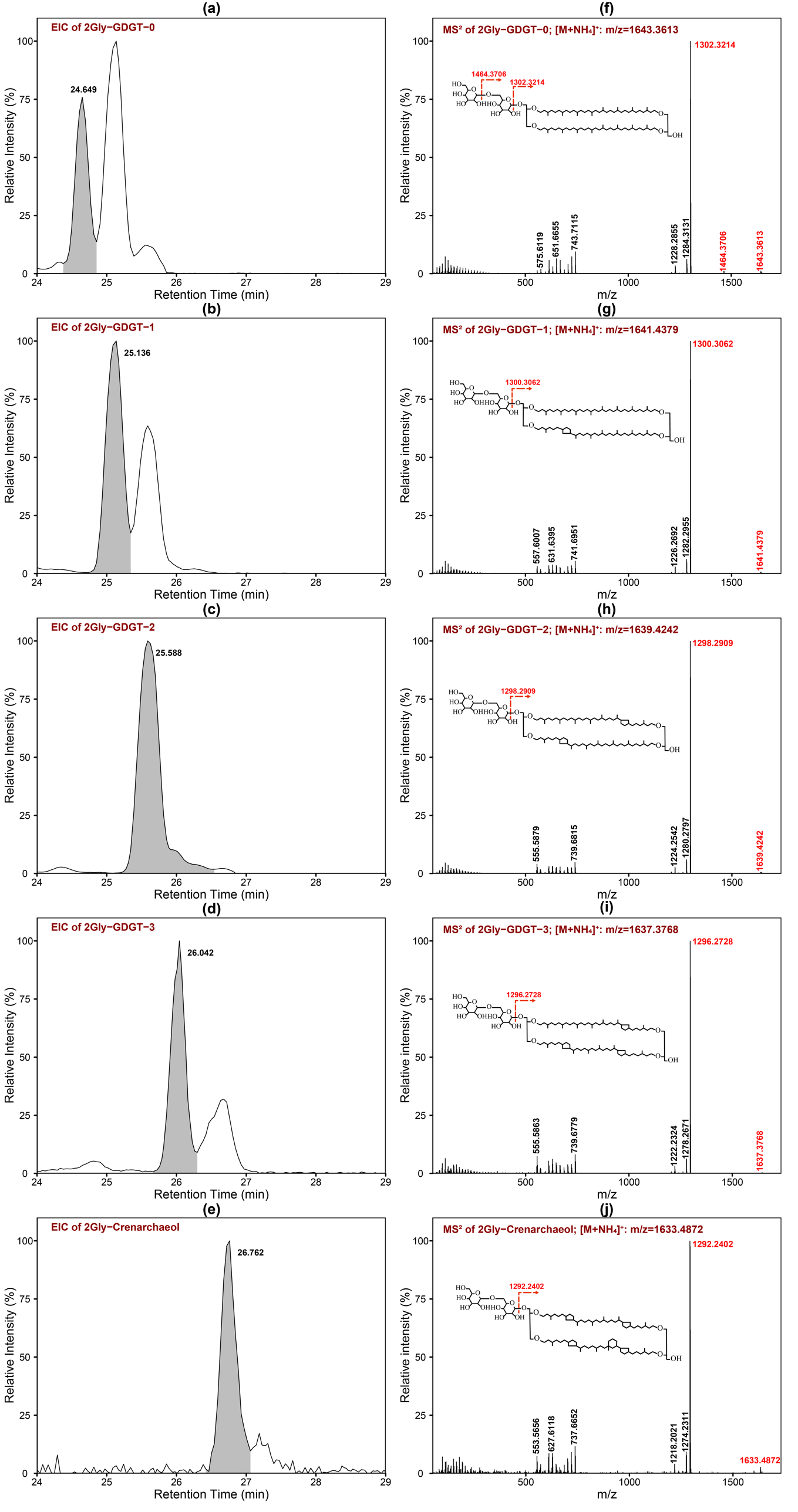


Figure S7. (a)-(e) Extract ion chromatograms (EICs, [M+H]^+^+[M+NH4]^+^+[M+Na]^+^) of 2Gly-GDGTs; (f)-(j) MS^2^ spectra of 2Gly-GDGTs, representative precursor ion and diagnostic product ions are highlight in red.


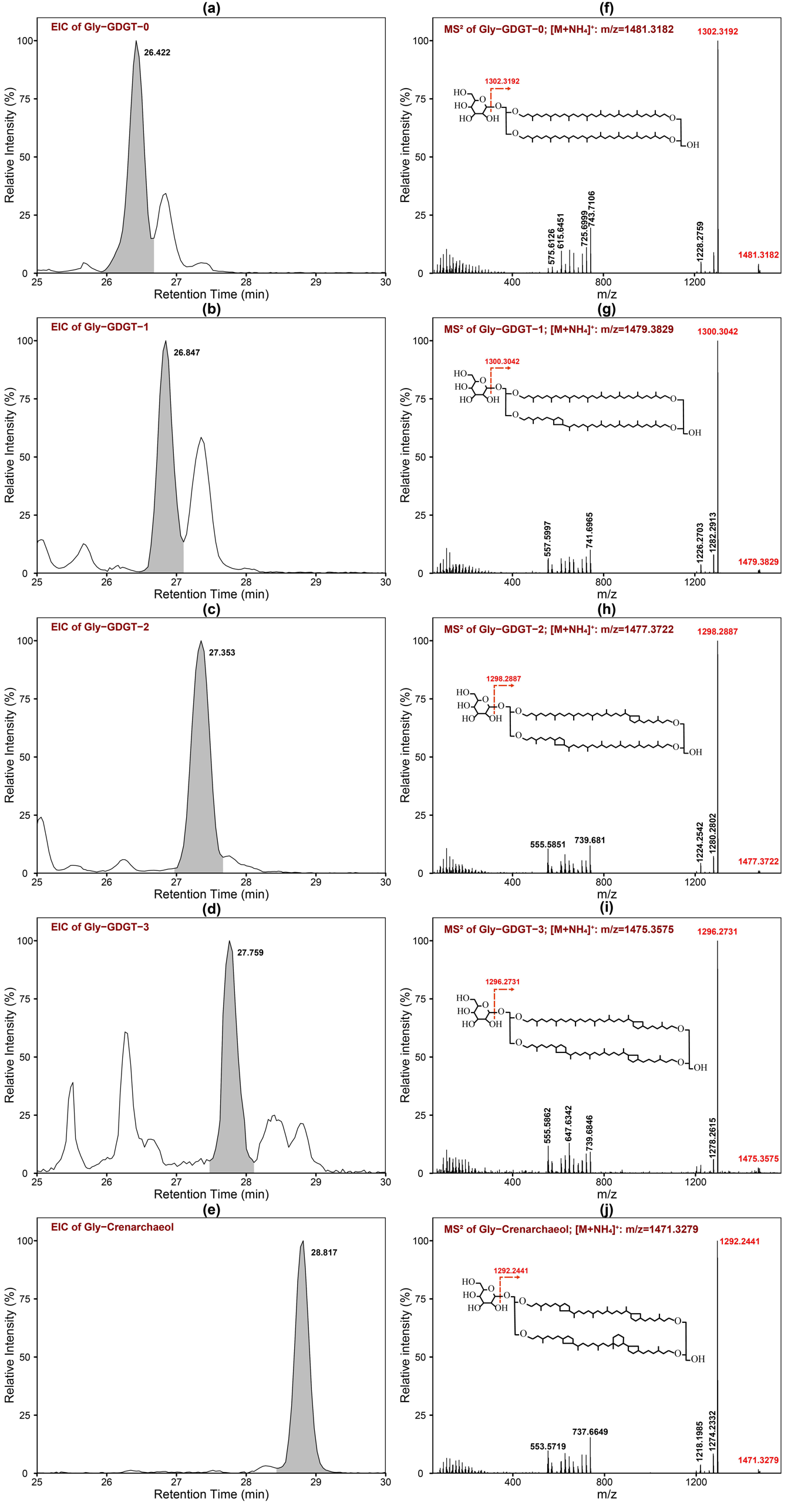


Figure S8. (a)-(e) Extract ion chromatograms (EICs, [M+H]^+^+[M+NH4]^+^+[M+Na]^+^) of Gly-GDGTs; (f)-(j) MS^2^ spectra of Gly-GDGTs, representative precursor ion and diagnostic product ions are highlight in red.


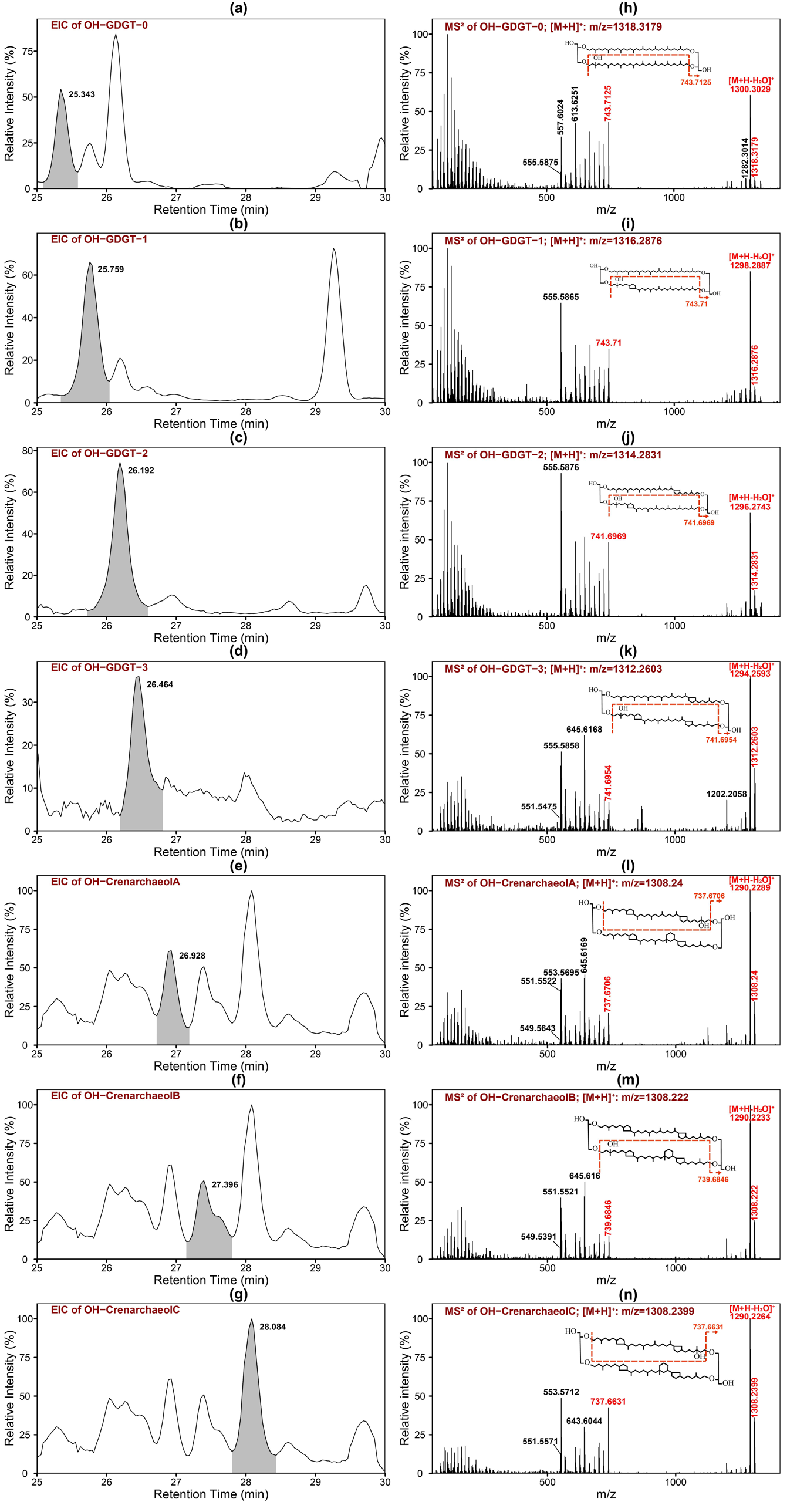


Figure S9. (a)-(g) Extract ion chromatograms (EICs, [M+H]^+^+[M+NH4]^+^+[M+Na]^+^) of OH-GDGTs; (h)-(n) MS^2^ spectra of OH-GDGTs, representative precursor ion and diagnostic product ions are highlight in red.


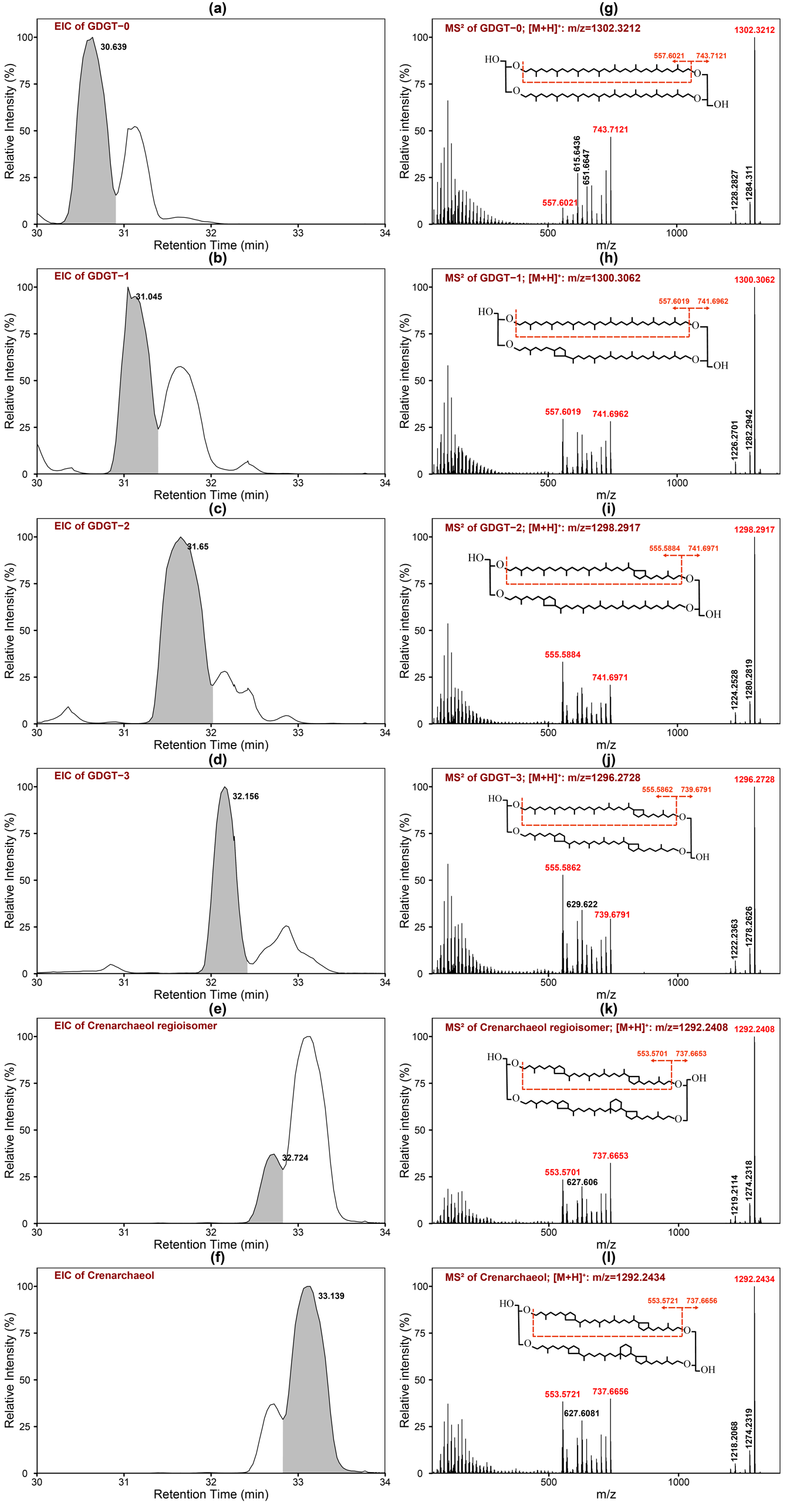


Figure S10. (a)-(f) Extract ion chromatograms (EICs, [M+H]^+^+[M+NH4]^+^+[M+Na]^+^) of GDGTs; (g)-(l) MS^2^ spectra of GDGTs, representative precursor ion and diagnostic product ions are highlight in red.


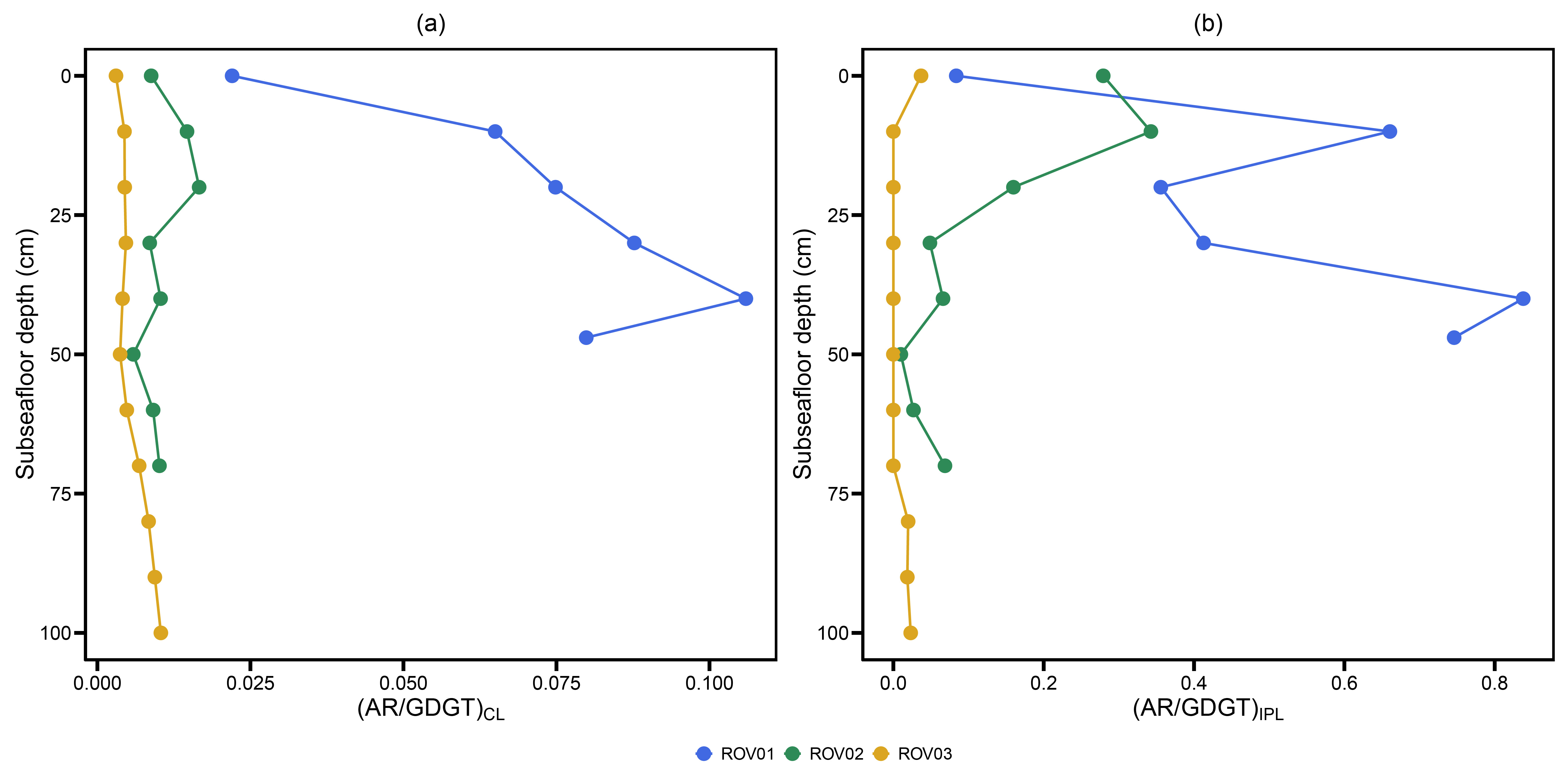


Figure S11. Ratios of (a) core diether lipids and tetraether lipids and (b) intact polar diether lipids and tetraether lipids.


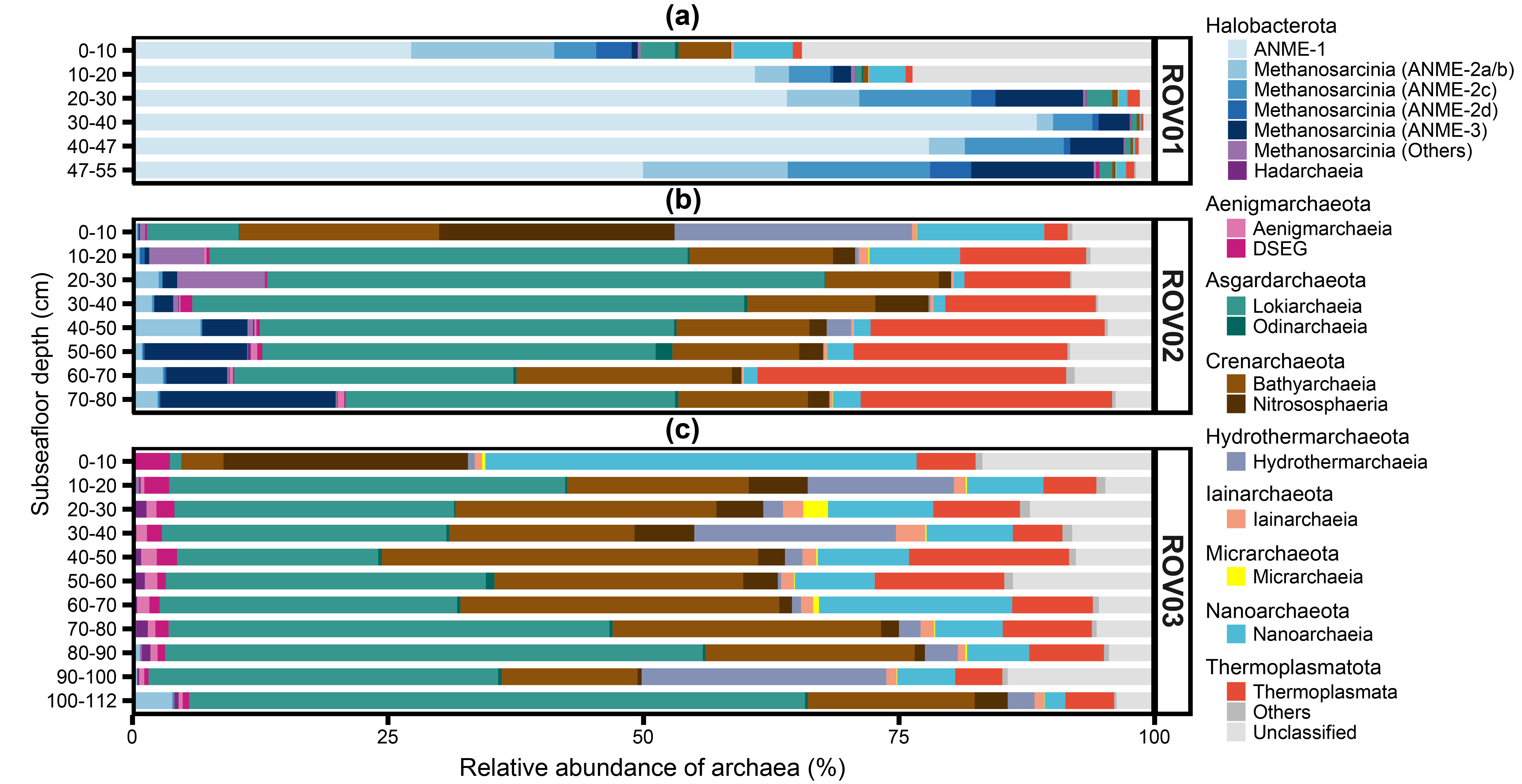


Figure S12. Archaeal composition based on 16S rRNA gene sequencing data in (a) ROV01, (b) ROV02, and (c) ROV03.


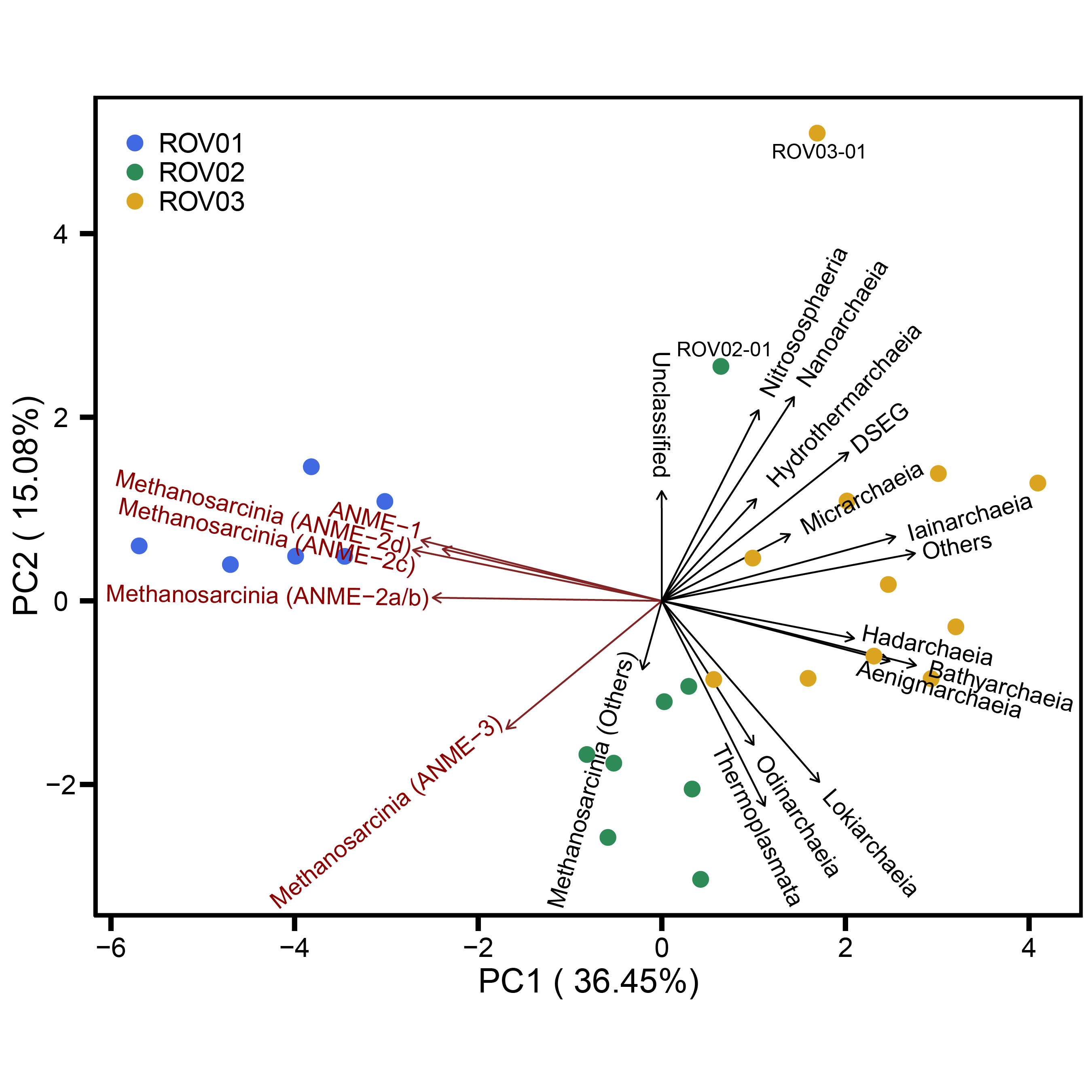


Figure S13. PCA plot based on class level archaea composition of samples from ROV01 (blue dots), ROV02 (green dots), and ROV03 (gold dots).
